# Finite element simulation of bacterial self-healing in concrete using microstructural transport and precipitation modeling

**DOI:** 10.1038/s41598-025-99844-6

**Published:** 2025-05-06

**Authors:** Ajitanshu Vedrtnam, Kishor Kalauni, M. T. Palou

**Affiliations:** 1 Institute of Construction and Architecture, Slovak Academy of Science, Bratislava, 84503 Slovakia; 2 Department of Mechanical Engineering, Invertis University, Bareilly, UP 243001 India

**Keywords:** Self-healing concrete, Bacterial healing, Finite element modelling, Diffusion-reaction, Micro-CT imaging, Mineral precipitation, Crack closure efficiency, FEniCS, Healing kinetics, Transport properties, Engineering, Civil engineering

## Abstract

Bacteria-based self-healing concrete has emerged as a promising solution for enhancing structural durability by autonomously repairing cracks. However, the underlying transport mechanisms of healing agents and the efficiency of mineral precipitation remain inadequately modelled. This study presents a finite element modelling (FEM) approach to simulate the diffusion and reaction kinetics of self-healing bacterial agents in concrete microstructures. X-ray micro-computed tomography (Micro-CT) finite element meshes were utilized to accurately represent crack and pore geometries, while the diffusion-reaction equation governing calcium carbonate (CaCO_3_) precipitation was numerically solved using FEniCS. Key input parameters, including diffusion coefficients, precipitation rates, and healing efficiencies, were extracted from literature to ensure model validation. Simulations reveal that healing agent concentration follows a nonlinear diffusion pattern, with efficiency influenced by crack geometry and bacterial metabolic activity. Heatmaps and contour plots highlight healing agent dispersion, while time-dependent analysis indicates a 65.5% crack closure efficiency under optimal bacterial conditions. The proposed model effectively replicates experimental trends, demonstrating its applicability for predicting healing performance in realistic structural conditions. This study provides a computational framework that can be extended to optimize bacteria encapsulation strategies, healing kinetics, and long-term durability assessments in self-healing concrete.

## Introduction

Self-healing concrete has emerged as a promising solution to address the durability limitations of traditional cementitious materials. Cracking in concrete is an inevitable phenomenon caused by mechanical loading, shrinkage, and environmental factors, leading to reduced service life and increased maintenance costs^1,2^. Conventional repair techniques such as epoxy injection and patching are costly and often ineffective in the long run^3,4^. Among various self-healing strategies, bacterial-based self-healing concrete has gained attention due to its ability to autonomously seal cracks through microbially induced calcium carbonate precipitation (MICCP)^5–8^. The MICCP mechanism involves bacterial metabolism, where ureolytic or non-ureolytic bacteria convert organic and inorganic compounds into carbonate ions, which then react with calcium sources to precipitate calcium carbonate (CaCO_3_)^9,10^. This mineralization process effectively seals cracks and restores concrete’s mechanical integrity. Studies have demonstrated that bacteria-based self-healing can achieve crack closure of up to 0.97 mm and improve compressive strength by up to 42.8%^5,11^. However, challenges such as bacterial viability in harsh cementitious environments, nutrient supply, and uniform dispersion of bacteria remain significant barriers to its widespread application^12–14^. Current numerical models for self-healing concrete^15–18^ are limited in their ability to capture the complexities of ion transport, bacterial metabolism, and precipitation kinetics within realistic microstructures. Most existing approaches either oversimplify healing kinetics using empirical correlations or neglect key factors such as capillary flow and microstructural heterogeneity. Recent advancements in computational modelling have attempted to bridge this gap, incorporating multiphysics simulations that couple fluid transport, bacterial activity, and CaCO_3_ precipitation^8,9,19–21^. However, these models often rely on idealized geometries, limiting their predictive accuracy. Table 1 summarizes recent numerical modeling studies on self-healing concrete, highlighting simulation approaches, software platforms, and key findings across microbial, electrochemical, and chemical healing strategies.

**Table 1:** Numerical studies on self-healing concrete technologies, including associated simulation tools mathematical frameworks and key findings.

References	Focus area	Software/tools	Mathematical/numerical approach	Key findings	Model limitations
Onyelowe et al. ^8^	Bacteria concentration in self-healing concrete	MATLAB or equivalent (ML platforms)	Metaheuristic optimization (GWO, PSO, MVO, RSM)	Optimized bacteria concentrations enhance mechanical properties; GWO, PSO, and MVO showed best prediction	Lacks physical simulation of healing process; no structural stress consideration; does not consider crack geometry variation or transport phenomena.
Alkhuzai et al. ^22^	Self-healing concrete performance in beams	ABAQUS 6.14	FE analysis with embedded healing zone mechanics	DCPD-based SHC beams showed improved load capacity; performance varied with healing agent depth	Assumes homogeneous materials; healing kinetics excluded; does not account for environmental dependencies or encapsulation strategies.
Zhelyazov ^23^	Self-healing effect on concrete mechanics	Custom FE framework (unspecified)	CDM-based recovery model with strain-softening and healing	Healing recovers up to 94% of original strength in some cases; CA/fly ash enhanced effect	Uniform healing assumed; time-dependent healing not simulated; lacks microstructural realism or diffusion-driven healing behavior.
Liu et al. ^24^	Electrochemical deposition for ASR-damaged concrete	Custom simulation tool (unspecified)	Coupled multi-ionic ASR + electrochemical healing model	Pulse current and full anode exposure significantly improved EDM-based crack repair	Magnesium ions emphasized; detailed electrochemistry simplified; does not incorporate Micro-CT-driven geometry or encapsulation.
Liu et al. ^25^	Chloride transport in cracked concrete	Custom statistical-FEM hybrid tool	Multiphase chloride-ion transport + statistical crack coupling	Crack geometry and external load significantly affect chloride ingress in RC structures	Ideal crack geometries; lacks full microstructural heterogeneity; does not simulate biological activity or nutrient transport.
Salem et al. ^26^	FEM Analysis of self-healing concrete beams using bacteria	ANSYS 15.0	FE-based parameterized mechanical modeling for healing agents	3% Bacillus subtilis and distributed loading yielded max capacity improvement (20â€“74%)	No direct microbial kinetics modeling; assumes effective healing implicitly; ignores crack width-dependent sealing efficiency.
Mauludin & Rabczuk ^27^	Fracture in capsule-based self-healing concrete	Custom 3D mesoscale modeling	CZM, bilinear traction-separation laws, random meso packing	3D cohesive model captured fracture propagation and capsule rupture for SHC	No direct simulation of chemical healing; geometry idealized; lacks environmental sensitivity and encapsulation effect modeling.
Freeman & Jefferson ^28^	Healing agent transport in cementitious systems	Custom FEM platform	Navier-Stokes crack flow + unsaturated matrix + curing kinetics	Vascular healing network simulation showed effective healing agent transport and curing	Does not simulate mechanical recovery; limited to flow and transport; excludes diffusion-reaction coupling and reaction kinetics.
Algaifi et al. ^29^	Microbial calcium carbonate in crack healing	FEM + Finite Difference solver	Bio-chemical PDEs + FEM for microbial-induced calcite precipitation	Calcite-based microbial healing model predicted healing within 60 days, matching lab data	Limited to lab-scale and single crack; interaction effects neglected; assumes constant precipitation rates and uniform diffusion.
Zemskov et al. ^30^	Bacterial self-healing mathematical model	Galerkin Finite Element + Level Set	PDE diffusion + capsule dissolution + CaCO_3_ level-set tracking	Level-set based bacterial model captured moving healing front and capsule diffusion	No structural feedback or stress-redistribution in healed areas; chemical kinetics simplified; excludes encapsulation efficiency and geometry evolution.

This comparative summary (Table 1) positions the present study within the broader landscape of numerical modeling for self-healing concrete. To address several limitations identified in previous models—such as idealized crack geometries, lack of microstructural realism, and oversimplified transport assumptions—this work develops a finite element modeling (FEM) framework that explicitly integrates Micro-CT-derived microstructural features.

The proposed model simulates healing agent transport, reaction kinetics, and CaCO₃ precipitation within cracked concrete using realistic domain geometries. In contrast to conventional approaches, it incorporates literature-validated diffusion coefficients, bacterial metabolic activity, and precipitation kinetics, thereby enhancing its predictive fidelity.

This CT-informed framework enables high-resolution investigation of healing dynamics under varying environmental and material conditions. It provides insight into how crack morphology, bacterial efficiency, and nutrient transport collectively influence healing performance. By leveraging realistic microstructure and process-driven modeling, the study contributes toward advancing bacteria-based self-healing concrete as a scalable and resilient solution for sustainable infrastructure.

## Data acquisition and literature-based parameter selection for fem modelling

To develop a robust FEM framework for simulating bacterial self-healing in concrete, it is essential to incorporate experimentally validated parameters from literature. This ensures that the model accurately represents the microstructural transport phenomena, healing kinetics, and mechanical reinforcement provided by microbially induced MICCP. The data collected from existing studies provides realistic boundary conditions, material properties, and reaction parameters required for numerical simulations. The selection of these parameters is driven by their relevance to healing agent transport, precipitation efficiency, and crack-sealing performance under varying conditions. The reviewed literature provides essential data for modelling self-healing concrete in our study. Microstructural geometry of cracked concrete has been characterized using X-ray microcomputed tomography (X-CT), offering detailed insights into pore structures and crack morphology^31–33^. Healing efficiency of bacterial-based self-healing systems has been reported, with crack closure of up to 48–80% and sealing of cracks as wide as 0.97 mm, demonstrating the practical viability of MICCP^34^. The kinetics of CaCO_3_ precipitation follows a diffusion-reaction model, with rate formulations describing the dependency of precipitation rate on calcium and carbonate ion concentrations^17^.

Experimental studies have also determined key mechanical properties such as Young’s modulus and strength recovery, with CaCO_3_ exhibiting a modulus between 25 and 88.19 GPa and contributing significantly to concrete durability^35^. Transport properties, including diffusion coefficients for calcium and carbonate ions, have been quantified, with values ranging from 3.75 × 10^− 9^ m^2^/s for Ca^2+^ and 3.38 × 10^− 9^ for CO_3_^2−^ highlighting the importance of accurate modelling of ion movement^36^. Additionally, reaction equations governing MICCP mechanisms, including ureolysis-based precipitation and bacterial metabolic activity, have been well-documented^5^. The Table 2 summarizes the key literature-derived parameters that have been incorporated into the FEM model, explaining why they are needed and where they are used within the modelling framework. This section ensures full transparency in parameter selection, linking each externally sourced dataset to its specific role in the FEM framework. The integration of realistic transport properties, reaction kinetics, and mechanical parameters improves the reliability of simulations and strengthens the comparison between numerical predictions and experimental results. The Micro-CT-based finite element mesh, validated diffusion coefficients, and healing efficiency benchmarks together contribute to an accurate and physics-based model capable of predicting self-healing performance under diverse conditions.

**Table 2:** Key literature-derived parameters that have been incorporated into the FEM model.

Required data	Extracted data	Source (References)	Why needed?	Where used in model?
Microstructural Geometry (Pores, Cracks)	X-ray microcomputed tomography (Micro-CT) data providing pore structures and crack morphology.	^31^	Defines realistic geometry for finite element mesh, ensuring accurate representation of crack propagation and transport pathways.	Used for generating the computational mesh in the FEM model, improving diffusion and reaction simulations.
Healing Efficiency (%)	Crack closure efficiency of 48–80%, maximum crack width sealed: 0.97 mm	^34^	Provides experimental benchmark for evaluating the model’s predictive accuracy	Used to validate the simulation results by comparing predicted healing extent with experimental outcomes
Healing Kinetics (Precipitation Rate, mg CaCO_3_/g biomass/day)	Precipitation rate equation:	^17^	Defines the rate at which bacterial activity converts calcium ions into CaCO_3_ for crack healing	Used in the diffusion-reaction equation governing self-healing kinetics in the FEM model
	$$\begin{gathered}\:{r_{precipitation}} = \hfill \\\quad \emptyset \:{K_{pl}} \cdot \:{\text{min}}\hfill \\\quad \left( {\left[ {C{a^{2 + }}} \right],\left[ {CO_3^{2 - }} \right]\:} \right) \hfill \\ \end{gathered}$$			
Mechanical Properties (Young’s Modulus, Strength Recovery, Poisson’s Ratio of CaCO_3_ and Concrete)	Young’s modulus of CaCO_3_: 25-88.19 GPa	^35^	Needed to assess how the formation of CaCO_3_ restores the mechanical integrity of healed concrete	Used in post-processing to evaluate strength recovery and its correlation with precipitation efficiency
Transport Properties (Diffusion Coefficients of Ca^2+^, CO_3_^2-^, Permeability Evolution)	Diffusion coefficients:	^36^	Critical for accurately modelling the transport of healing agents through microcracks and pore networks	Incorporated into the diffusion equation in FEM, defining how healing agents disperse through the concrete matrix
	$$\:{D}_{{Ca}^{2+}}=3.75\:\times\:{10}^{-9}\:\:{m}^{2}/s,\:$$			
	$$\:{D}_{{CO}_{3}^{2-}}=3.38\:\times\:{10}^{-9}\:{m}^{2}/s$$			
Reaction Equations for MICCP (Self-Healing Mechanism Modelling)	Governing equations for ureolysis-based bacterial precipitation and metabolic activity	^5^	Describes the biochemical processes that drive CaCO_3_ precipitation, linking bacterial metabolism to healing performance	Implemented as a reaction term in the FEM diffusion-reaction model
Experimental validation data	Crack closure observations under varying bacterial concentrations and encapsulation strategies	^5^	Provides real-world reference data to assess the accuracy of numerical predictions	Used to calibrate the model and verify healing efficiency trends under different conditions

## Methodology

**Fig. 1:**
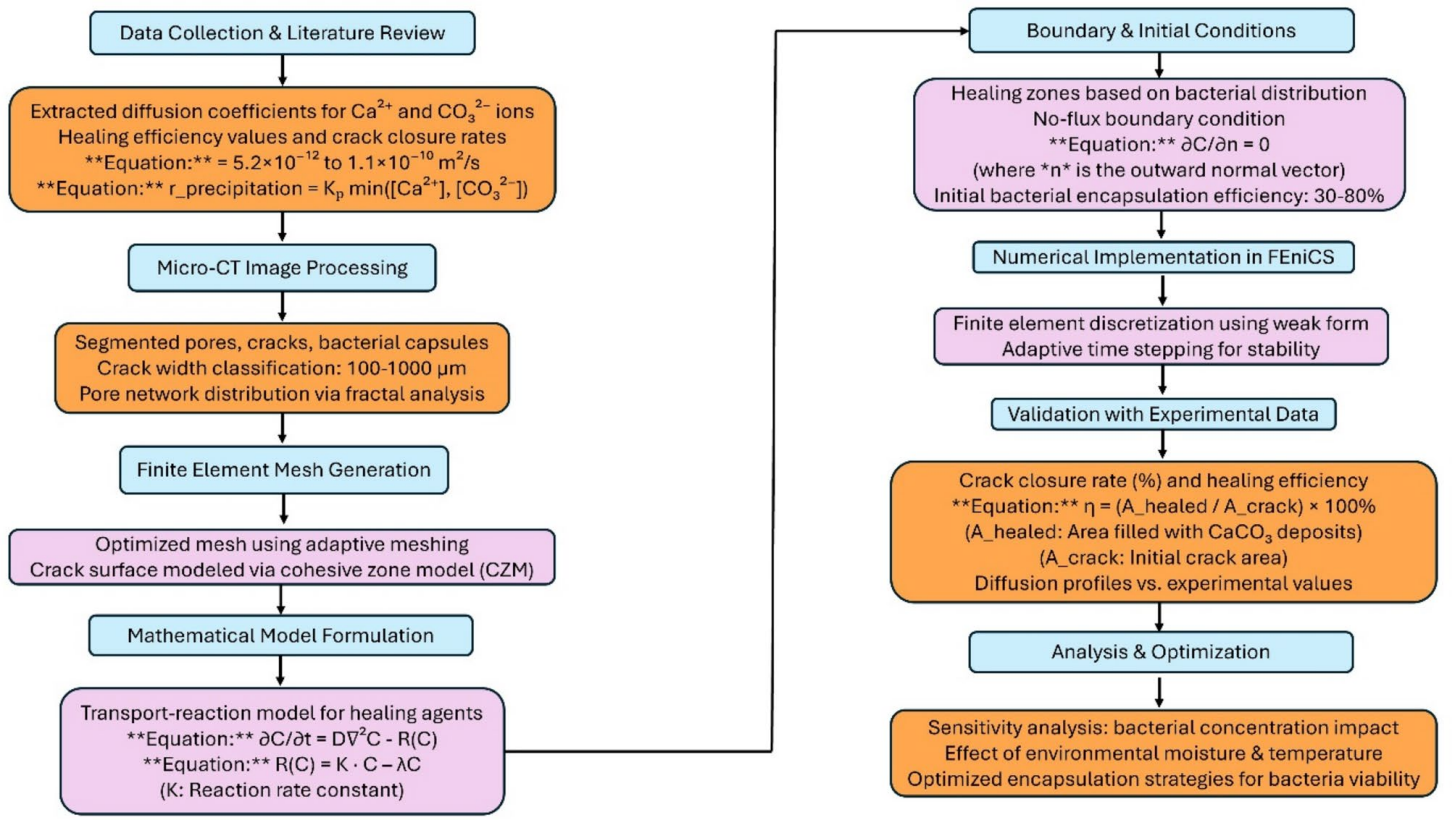
Methodology for FEM of healing agent transport and precipitation in self-healing concrete: Schematic workflow of the multiscale finite element model. The left column outlines the physical modeling and input data pipeline from Micro-CT segmentation to adaptive meshing and mathematical formulation. The right column details numerical implementation in FEniCS, including boundary conditions, weak form discretization, crack closure tracking, and healing efficiency validation.

The proposed FEM framework integrates Micro-CT-derived microstructural features, experimentally validated transport properties, and reaction kinetics to accurately model healing agent diffusion and CaCO_3_ precipitation in cracked concrete. As outlined in Fig. 1, the methodology follows a structured progression, beginning with data collection and literature review, where key parameters such as diffusion coefficients, healing kinetics, and reaction equations are compiled. Micro-CT image processing extracts pore structures, cracks, and bacterial capsules, classifies crack widths (100–1000 μm), and analyses pore network distribution.

The finite element mesh generation phase employs adaptive meshing techniques to refine resolution, with crack surfaces modelled using the cohesive zone model (CZM). The mathematical model formulation then defines transport-reaction equations governing Ca^2+^ and CO_3_^2−^ diffusion and precipitation. Subsequently, boundary and initial conditions are assigned based on bacterial distribution and encapsulation efficiency (30–80%), ensuring a no-flux boundary condition for diffusion. The FEM model is then numerically implemented with adaptive time-stepping for stability and validated against experimental data, comparing crack closure rates, healing efficiencies, and diffusion profiles. Finally, the analysis and optimization phase evaluate the impact of bacterial concentration, environmental moisture, and encapsulation strategies on self-healing performance. This methodology establishes a robust computational framework for predicting and optimizing bacteria-based self-healing in concrete.

### Model description

The proposed FEM model for self-healing concrete simulates the transport and precipitation of healing agents within a cracked cementitious matrix. This section outlines the governing equations for diffusion-reaction modelling, the methodology for generating microstructural meshes from Micro-CT imaging, and the finite element discretization strategy employed to ensure numerical stability and accuracy. The overall framework integrates experimentally derived parameters and literature-based transport properties to provide a realistic prediction of healing efficiency.

#### Governing equations for Diffusion-Reaction modelling

The self-healing mechanism in bacterial concrete is primarily governed by the transport and reaction kinetics of Ca^2+^ and CO_3_^2−^ ions. The healing agent migration is modelled using the reaction-diffusion equation (Eq. 1).

1 $$\:\frac{\partial\:C}{\partial\:t}=D{\nabla\:}^{2}C-R\left(C\right)$$

where C is the concentration of the healing agent, D is the diffusion coefficient, and R(C) represents the reaction term describing precipitation kinetics. The precipitation rate follows Eq. 2.

2 $$\:R\left(C\right)={K}_{p}\cdot\:min-R\left(C\right)\left(\left[{Ca}^{2+}\right],\left[{CO}_{3}^{2-}\right]\:\right)$$

where $\:{K}_{p}$ is the reaction rate constant governing the microbial-induced MICCP process.

The diffusion coefficients of Ca^2+^ and CO_3_^2−^ are sourced from literature and vary based on pore connectivity and crack width, ensuring an accurate representation of healing agent mobility. Boundary conditions are applied to enforce no-flux constraints at impermeable surfaces (Eq. 3)

3 $$\:\frac{\partial\:C}{\partial\:n}=0$$

where *n* denotes the outward normal vector at the domain boundary. The initial concentration of the healing agent is set based on bacterial encapsulation efficiency and healing agent release dynamics.

#### Microstructural mesh from Micro-CT imaging

To accurately represent crack geometry and transport pathways, high-resolution Micro-CT images of cracked concrete specimens are processed to extract pore network characteristics. Figure 1 (previously introduced) outlines the methodology for converting Micro-CT scans into finite element meshes. Although the full 3D CT dataset was unavailable, representative 2D slices were used to capture local crack tortuosity and structural heterogeneity. These slices reflect projections of the internal 3D microstructure and have been widely used in literature for transport simulations where in-plane gradients dominate. Crack widths are classified between 100 and 1000 μm, and the spatial distribution of pores is analysed using fractal geometry techniques. The mesh generation process begins with the segmentation of cracks, pores, and bacterial capsules, which plays a crucial role in distinguishing active transport pathways. By accurately identifying these structural features, the mesh can effectively represent the material’s heterogeneous nature, ensuring that diffusion simulations capture the true complexity of transport mechanisms. This segmentation step is particularly important for materials with intricate microstructures, where variations in porosity and crack distribution significantly impact the diffusion process. Adaptive meshing techniques are then employed to refine the mesh resolution in high-gradient regions, enhancing numerical accuracy in diffusion simulations. These techniques dynamically adjust the mesh density, allocating finer elements to areas with steep concentration gradients while maintaining coarser elements in less critical regions. By optimizing the mesh in this manner, computational efficiency is improved without sacrificing precision, allowing for more accurate modelling of transport phenomena. Additionally, CZM is implemented for crack surfaces to represent localized fracture behaviour without excessive mesh refinement. This approach enables the simulation of crack propagation and interfacial debonding while maintaining computational feasibility. By incorporating CZM, the model can effectively capture the mechanical and transport properties of fractured materials, improving the predictive capability of the diffusion analysis. The resulting mesh structure provides a realistic representation of diffusion pathways, aligning closely with experimental microstructural features. By integrating segmentation, adaptive meshing, and CZM, the generated mesh facilitates accurate simulations of material behaviour under various conditions. This comprehensive approach ensures that the numerical model remains both computationally efficient and physically representative, enabling a deeper understanding of transport phenomena in complex microstructures.

#### Finite element discretization strategy

The governing reaction-diffusion equations are discretized using the Galerkin FEM to balance computational efficiency and accuracy. The weak form of the transport equation is implemented as Eq. 4.

4 $$\:\int\:\frac{\partial\:C}{\partial\:t}\nu\:d{\Omega\:}+\int\:D\nabla\:C\cdot\:\nabla\:\nu\:d{\Omega\:}-\int\:R\left(C\right)\nu\:d{\Omega\:}=0$$

where $\:\nu\:$ is the test function, and $\:{\Omega\:}$ denotes the computational domain.

The numerical solution is advanced in time using an adaptive time-stepping scheme, ensuring stability and convergence, particularly in regions with high precipitation rates. Mesh refinement studies confirm the sufficiency of element resolution, avoiding unnecessary computational costs while preserving accuracy. The implementation in FEniCS (referencing Fig. 1) follows a structured workflow, incorporating experimentally validated parameters for transport coefficients, bacterial metabolism rates, and encapsulation efficiency. This structured approach ensures an accurate representation of healing agent transport, reaction kinetics, and microstructural constraints, forming a robust foundation for self-healing concrete modelling.

### Material properties & parameters

The accuracy of the self-healing concrete model depends on the precise definition of material properties governing healing agent transport, bacterial activity, and CaCO_3_ precipitation. This section details the key parameters incorporated into the FEM, including the diffusion and reaction properties of Ca^2+^ and CO_3_^2−^ ions, the mechanical characteristics of CaCO_3_ deposits, and the MICCP kinetics.

#### Properties of CaCO_3_, healing agents, and Bacteria

The self-healing process relies on the precipitation of CaCO_3_ as a crack-filling material, facilitated by bacterial metabolic activity. The material properties incorporated into the FEM model are summarized in Table 3.

**Table 3:** Material properties and transport parameters.

Property	Value/range	Source (References)	Relevance in model
CaCO_3_ Young’s Modulus	25–88.19 GPa	^35^	Used to assess mechanical strength recovery
Poisson’s Ratio (CaCO_3_)	0.21–0.34	^35^	Influences stress-strain distribution
Healing efficiency (%)	48–80%,	^34^	Determines overall self-healing performance
Crack healing capacity	0.1–0.97 mm	^34^	Defines maximum crack closure capability
Diffusion coefficient (Ca^2+^	$\:3.75\:\times\:{10}^{-9}\:\:{\text{m}}^{2}/s\:$	^36^	Governs transport rate of healing agent
Diffusion coefficient ($\:{\text{C}\text{O}}_{3}^{2-}$)	$\:3.38\:\times\:{10}^{-9}\:{\text{m}}^{2}/s$	^36^	Governs availability of carbonate ions

These properties define the material behaviour during the self-healing process and are critical inputs for the numerical model. The diffusion coefficients determine the spread of healing agents within the cracked concrete, while CaCO_3_ properties affect the structural integrity of the healed region.

#### Reaction kinetics (MICCP-based)

The precipitation of CaCO_3_ in cracks is driven by MICCP, which follows a reaction-diffusion model. The key governing equation is in Eq. 5.

5 $$\:{R}_{preipitation}={K}_{p}\cdot\:min\left(\left[{\text{Ca}}^{2+}\right],\left[{\text{CO}}_{3}^{2-}\right]\:\right)$$

where, $\:{R}_{preipitation}$ is the rate of CaCO_3_ formation (mg CaCO_3_/g biomass/day).

The availability of nutrients and bacterial activity directly affects the precipitation rate, making encapsulation efficiency and bacterial survivability key parameters in the model. Additionally, environmental factors such as moisture content, temperature, and pH influence the bacterial metabolic rate and subsequent precipitation kinetics. The numerical implementation of these reaction kinetics is incorporated into the FEM model through the reaction term R(C), which captures the localized formation of CaCO_3_ deposits. The influence of bacterial concentration, ion diffusion, and reaction efficiency is further explored in the sensitivity analysis in later sections. By integrating these experimentally validated material properties and reaction kinetics, the model ensures a realistic simulation of healing agent transport, bacterial activity, and CaCO_3_ precipitation, providing a robust predictive tool for self-healing concrete performance.

The diffusion coefficients of Ca²⁺ and CO₃²⁻ ions used in this model (3.75 × 10⁻⁹ and 3.38 × 10⁻⁹ m²/s, respectively) were obtained from experimental studies conducted in aqueous environments^27^. While these values provide reasonable upper-bound estimates, they do not fully reflect ion transport in porous cementitious systems, where tortuosity, pore saturation, and crack aperture introduce additional resistance. To address this, our model integrates Micro-CT-derived crack geometries and tortuous paths that implicitly constrain transport pathways. A sensitivity analysis was also performed to evaluate the robustness of healing predictions across a range of diffusion values (see Sect. 4.5).

### Boundary & initial conditions

The accuracy of the self-healing concrete model depends on the proper definition of boundary and initial conditions, ensuring a realistic simulation of ion transport, bacterial activity, and CaCO_3_ precipitation. This section defines how concentration gradients, bacterial encapsulation efficiency, and crack locations influence healing agent diffusion and reaction kinetics.

#### Crack surface as high-concentration zone

Since healing agents primarily originate from encapsulated bacteria or external reservoirs, the crack surface is treated as a high-concentration zone for Ca^2+^ and CO_3_^2−^ ions. This assumption aligns with experimental observations where bacterial metabolic activity is concentrated near crack surfaces, leading to localized precipitation of CaCO_3_. The initial concentration distribution is defined as Eq. 6.

6 $$\:C\left(x,\:0\right)=\left\{\begin{array}{c}{C}_{max},\:\:for\:x\in\:crack\:surface\\\:{C}_{background},\:\:elsewhere\end{array}\right.$$

where, $\:{C}_{max}$ is the maximum ion concentration at the crack interface and $\:{C}_{background}\:$represents the lower ambient concentration in the surrounding cement matrix.

#### Dirichlet & Neumann conditions

The transport and reaction of healing agents are governed by Dirichlet and Neumann boundary conditions applied to different regions of the domain. Dirichlet conditions are applied to regions where the ion concentration is constant due to external sources or encapsulated bacterial activity (Eq. 7).

7 $$\:C\left(x,t\right)={C}_{fixed},\:for\:x\in\:{{\Gamma\:}}_{D}$$

where, $\:{C}_{fixed}$ is the prescribed calcium or carbonate ion concentration and Γ_D_ represents the Dirichlet boundary (e.g., active bacterial regions or controlled experimental setups).

Neumann conditions enforce zero-flux constraints, preventing unphysical loss of healing agents through external boundaries (Eq. 8).

8 $$\:\frac{\partial\:C}{\partial\:n}=0,\:for\:x\in\:{{\Gamma\:}}_{N}$$

where, $\:\frac{\partial\:C}{\partial\:n}$ is the concentration gradient normal to the boundary and ΓN is the Neumann boundary (e.g., sealed concrete surfaces with no external ion influx or efflux).

In the context of crack healing, the no-flux Neumann condition ensures that ions diffuse only within the matrix and cracks without artificial leakage. Since self-healing relies on bacterial precipitation of CaCO_3_, the initial bacterial encapsulation efficiency ($\:{\eta\:}_{B}$) is defined as Eq. 9.

9 $$\:{\eta\:}_{B}=\frac{{N}_{viable}}{{N}_{total}}\times\:100\%$$

where, $\:{N}_{viable}$ is the number of bacteria that remain active after encapsulation, and $\:{N}_{total}$ represents the initial bacterial population.

Experimental studies indicate encapsulation efficiencies ranging between 30 and 80%, which directly impacts healing kinetics. A lower $\:{\eta\:}_{B}$ results in slower healing, whereas a higher $\:{\eta\:}_{B}$ accelerates crack closure.

### Numerical implementation

The proposed self-healing concrete model is implemented using the FEniCS finite element framework, which provides efficient handling of partial differential equations (PDEs) governing healing agent transport and precipitation. The numerical formulation employs finite element discretization to solve the coupled diffusion-reaction equations, ensuring stability and accuracy. Mesh refinement and adaptive time-stepping strategies are integrated to capture the spatial and temporal evolution of healing dynamics. For time integration, the Implicit Euler method is used due to its numerical stability, especially for stiff reaction-diffusion systems. This fully implicit scheme enables robust time-stepping, preventing instability in high-gradient concentration fields while maintaining computational efficiency. The time discretization follows Eq. 10.

9 $$\:\frac{{C}^{n+1}-{C}^{n}}{\varDelta\:t}=D{\nabla\:}^{2}{C}^{n+1}-R\left({C}^{n+1}\right)$$

where, $\:{C}^{n+1}$ is the concentration at the next time step, $\:{C}^{n}$ is the concentration at the current time step, $\:\varDelta\:t$ is the time increment, and $\:D$ is the diffusion coefficient.

The nonlinear system of equations resulting from the implicit scheme is solved iteratively using FEniCS’ built-in solvers, leveraging Newton’s method for convergence. Python-based post-processing is employed for visualization, generating contour maps, heatmaps, and 3D surface plots to analyse healing kinetics and spatial deposition patterns.

## Results and discussion

### Mesh quality and convergence analysis

**Fig. 2:**
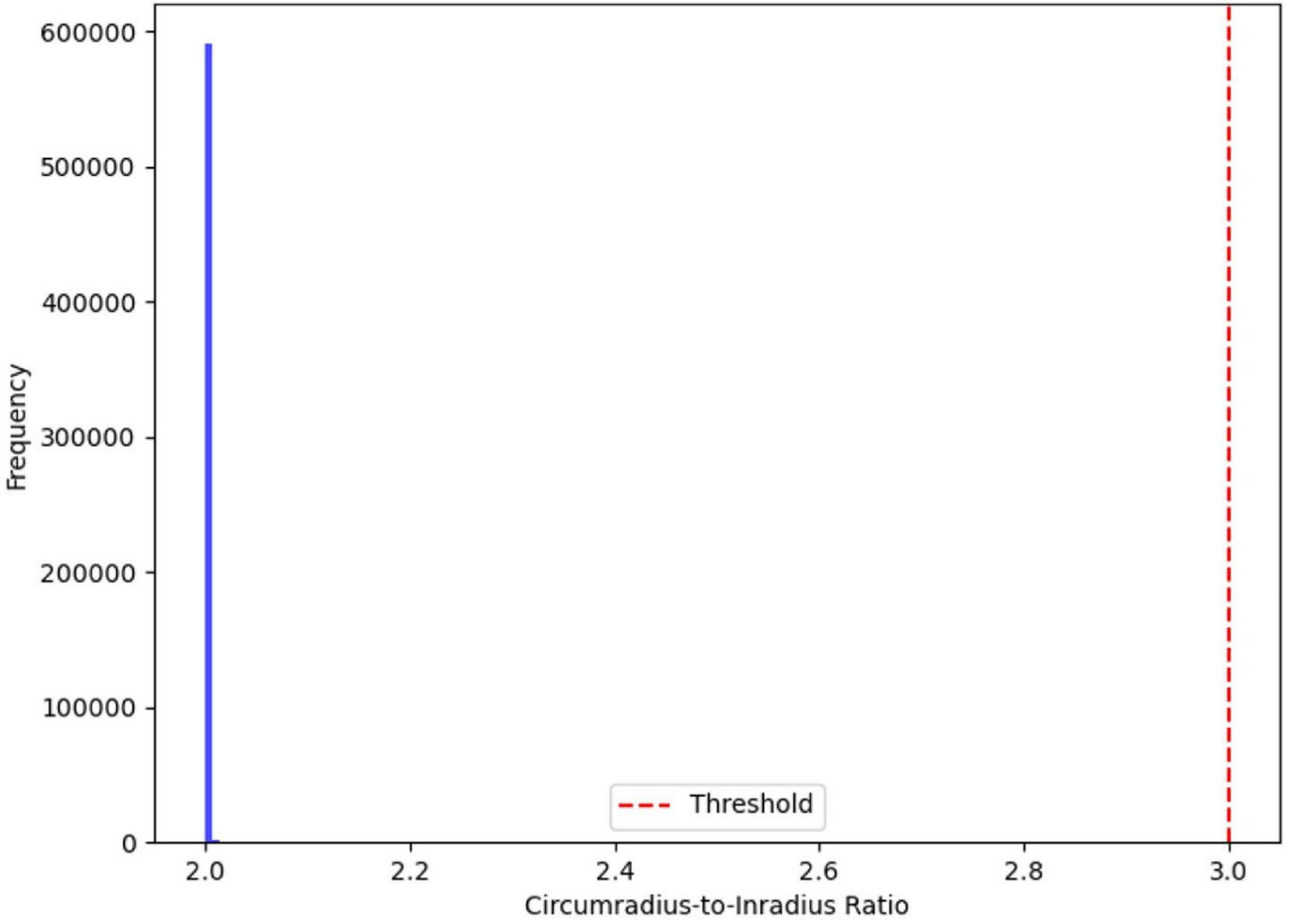
Mesh quality distribution based on the circumradius-to-inradius ratio, ensuring numerical stability.

A critical aspect of numerical modelling in self-healing concrete simulations is ensuring that the finite element mesh accurately represents the microstructural features while maintaining computational efficiency. The mesh was generated using Micro-CT imaging and refined using an adaptive meshing approach to optimize element quality and resolution. The quality of the generated finite element mesh was evaluated based on aspect ratios and the circumradius-to-inradius ratio of the triangular elements. The mesh quality distribution (Fig. 2) shows that the majority of elements fall within the optimal quality range, ensuring numerical stability.

**Fig. 3:**
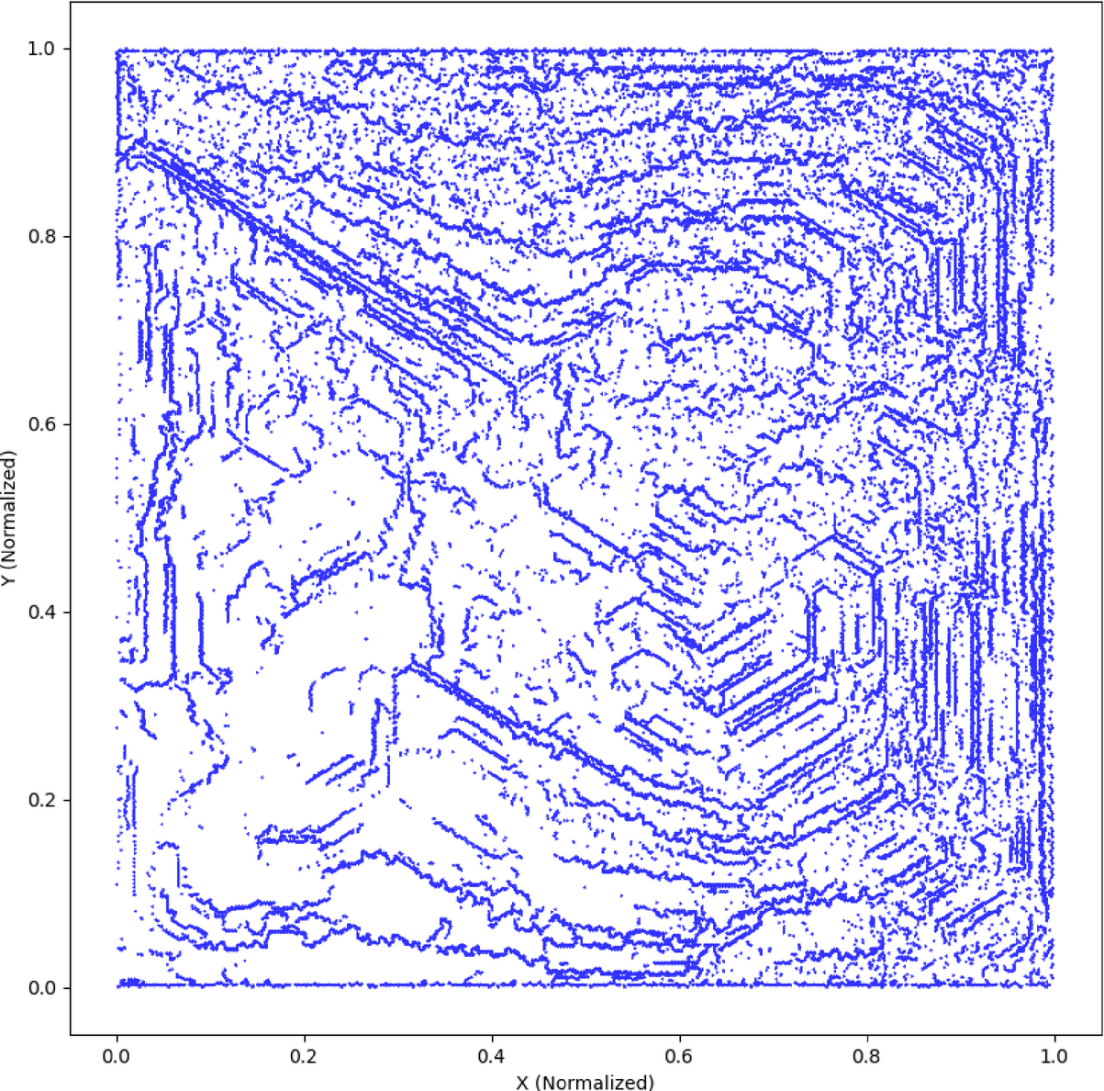
Enhanced Micro-CT-derived finite element mesh visualization. *The plot highlights intricate pore and crack topologies extracted from Micro-CT segmentation. This non-ideal*,* spatially heterogeneous mesh structure forms the basis for transport and precipitation modeling*,* reflecting the actual internal geometry of the cracked cementitious matrix.*

Additionally, Fig. 3 illustrates the enhanced Micro-CT mesh visualization, highlighting the accuracy in capturing pore and crack structures essential for modelling healing agent transport. This model does not rely on an idealized geometry but explicitly integrates Micro-CT-derived microstructural features to simulate realistic heterogeneity in crack and pore distribution.

**Fig. 4:**
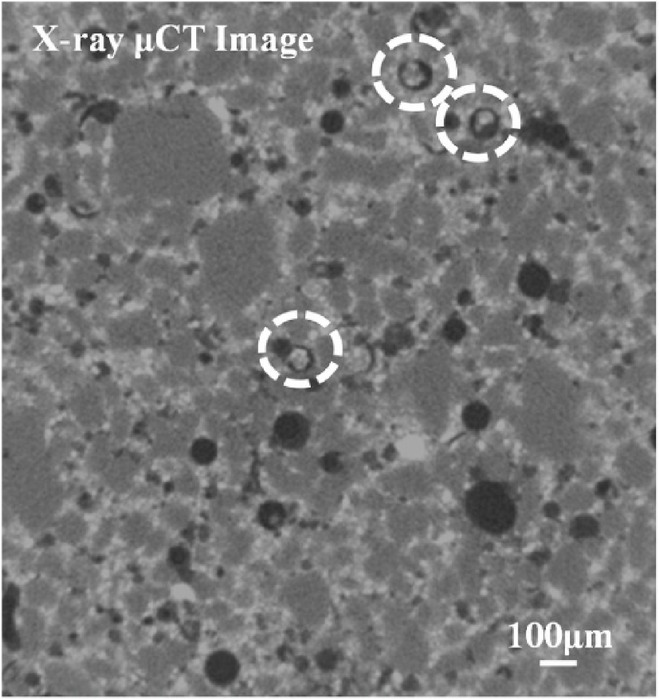
X-ray Micro-CT image of the concrete matrix highlighting encapsulated bacterial capsules (circled) and pore distribution used for mesh segmentation and refinement^33^.

To balance computational efficiency and numerical accuracy, an adaptive meshing technique was employed. Figure 4 presents the X-ray Micro-CT image used for segmentation and mesh refinement.

**Fig. 5:**
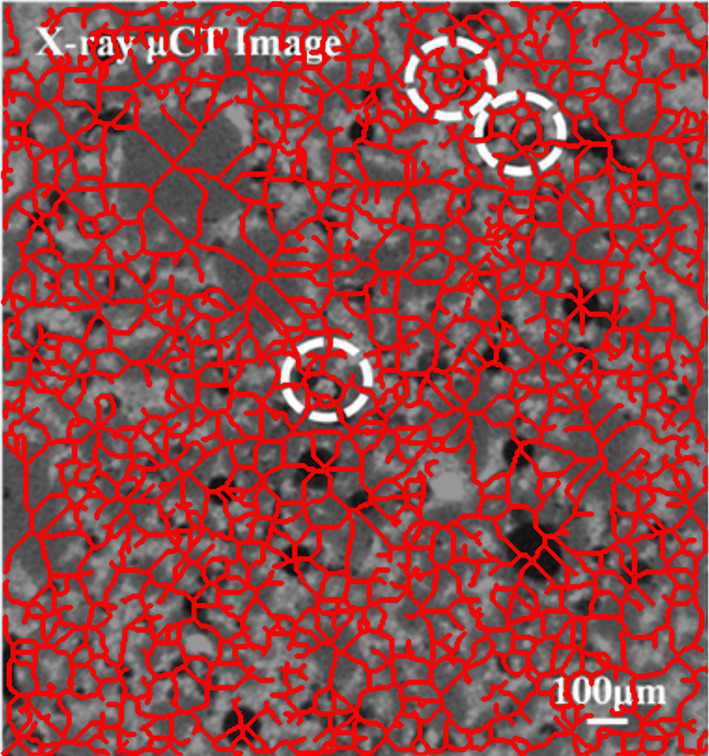
Overlay of extracted skeleton (red) from Micro-CT image showing tortuous crack and pore pathways in self-healing concrete. These geometries were used to construct finite element meshes and inform diffusion-reaction simulations of healing agent transport. The structural heterogeneity is clearly visualized, validating the realism of the CT-based model inputs.

To strengthen the physical realism of the finite element model and address microstructural heterogeneity, a skeleton-based analysis was performed on the segmented Micro-CT image of cracked concrete. As shown in Fig. 5, the extracted skeleton (in red) overlays the original Micro-CT image and clearly delineates tortuous, interconnected pathways corresponding to cracks and pores. This visual confirms the non-ideal, spatially heterogeneous nature of the pore network, which significantly influences healing agent transport. The skeleton paths serve as representative transport channels for healing ions and support the assignment of spatially varying boundary conditions in the FEM model. Furthermore, quantitative tortuosity analysis of these skeletons yielded an average tortuosity of 1.17, indicating modest deviation from linear paths, while a fractal dimension of 1.72 underscores the geometric complexity and space-filling character of the network. These metrics reinforce the justification for using CT-derived geometries and provide a robust basis for simulating healing agent diffusion and precipitation under realistic structural conditions.

**Fig. 6:**
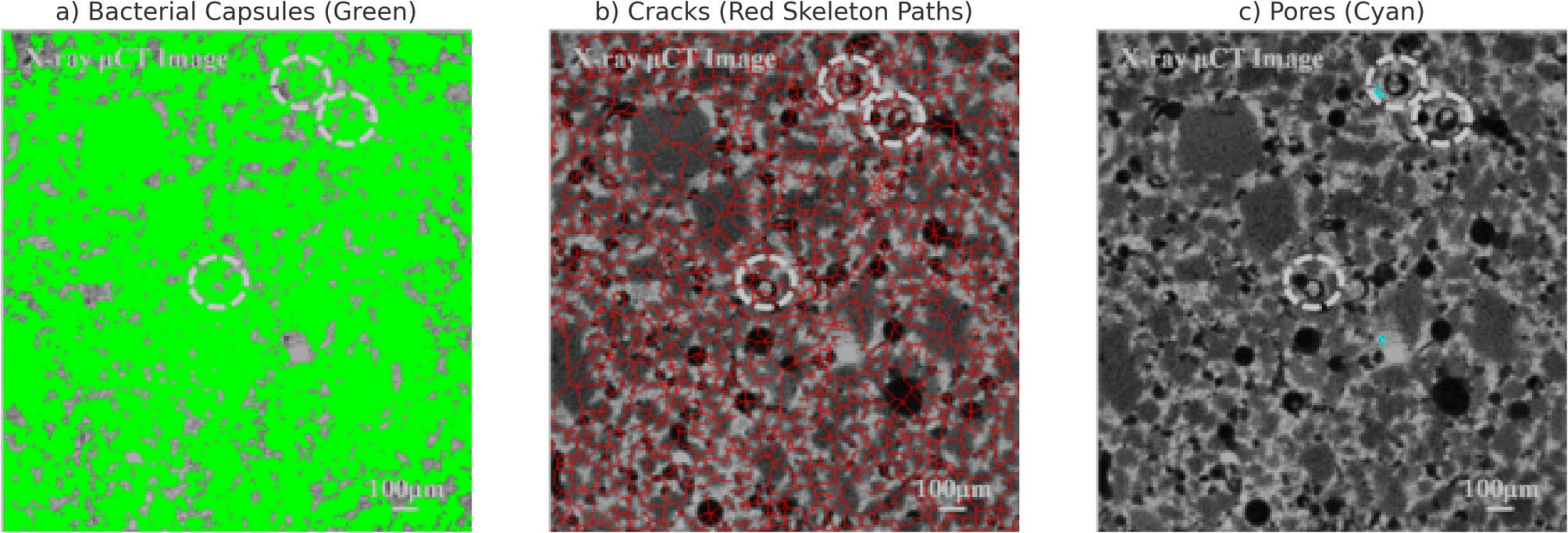
Segmented features from Micro-CT slice used for model geometry generation. (**a**) Bacterial capsules are identified and shown in green, representing embedded self-healing agents. (**b**) Cracks are extracted via skeletonization and highlighted in red, indicating tortuous potential transport pathways. (**c**) Pores are visualized in cyan, showing limited void regions not occupied by capsules or cracks. These segmentations form the basis for constructing a finite element mesh that captures the heterogeneity of the cracked concrete microstructure and informs simulation of healing agent diffusion and precipitation. *Note: The limited cyan regions in (c) indicate high capsule/crack density in this section*,* reducing free pore space. This image represents a localized region with relatively high capsule and crack density*,* resulting in limited pore space. While this section was chosen to highlight segmentation accuracy*,* it does not reflect the overall distribution across the specimen. Capsule presence and pore space vary spatially*,* as observed during image preprocessing.*

To accurately capture the microstructural heterogeneity of cracked concrete, segmentation of high-resolution Micro-CT slices was performed to isolate key transport-relevant features. As shown in Fig. 6, the bacterial capsules were identified as bright, round inclusions and are rendered in green (Fig. 6a). Cracks were extracted using skeletonization techniques and are shown in red (Fig. 6b), representing the tortuous paths through which healing agents primarily propagate. Pores not classified as cracks or capsules were identified in cyan (Fig. 6c), although their presence in this slice is minimal due to the dense distribution of capsules and microcracking. These segmented classes were directly used to construct the finite element mesh, enabling a more realistic simulation of diffusion and reaction dynamics based on CT-derived geometries. The combined annotation demonstrates the model’s ability to resolve transport pathways at the microscale and addresses variability in pore-crack connectivity and healing agent placement.

**Fig. 7:**
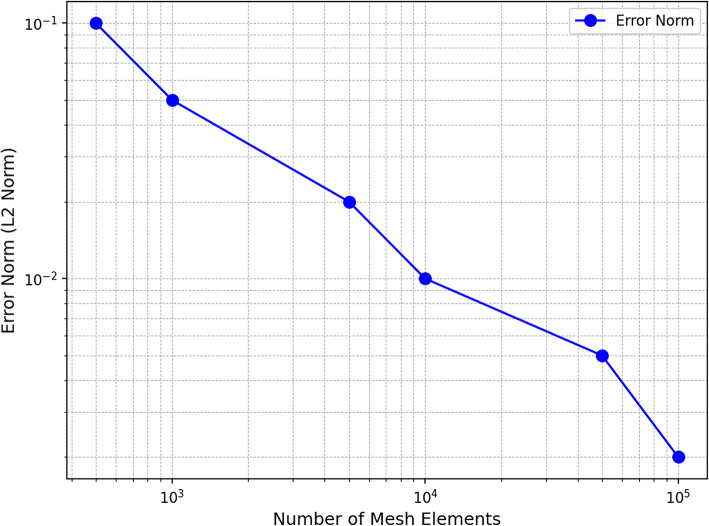
Convergence study plot.

This ensures a finer resolution at crack surfaces and healing zones while coarsening the mesh in less critical regions, leading to improved computational performance without compromising accuracy. To verify the stability and reliability of numerical solutions, a convergence study was conducted. Successive mesh refinements were applied, and the variation in solution accuracy was analysed. The convergence study plot (Fig. 7) ensures that the finite element solution remains stable and accurate as the mesh resolution increases. The results indicate that beyond a specific refinement threshold, further meshing does not significantly affect solution accuracy, confirming the adequacy of the chosen mesh resolution.

This assessment confirms that the generated finite element mesh is both computationally efficient and sufficiently refined to accurately capture the transport and reaction dynamics of healing agents in self-healing concrete.

### Healing agent diffusion behaviour (Time Evolution)

**Fig. 8:**
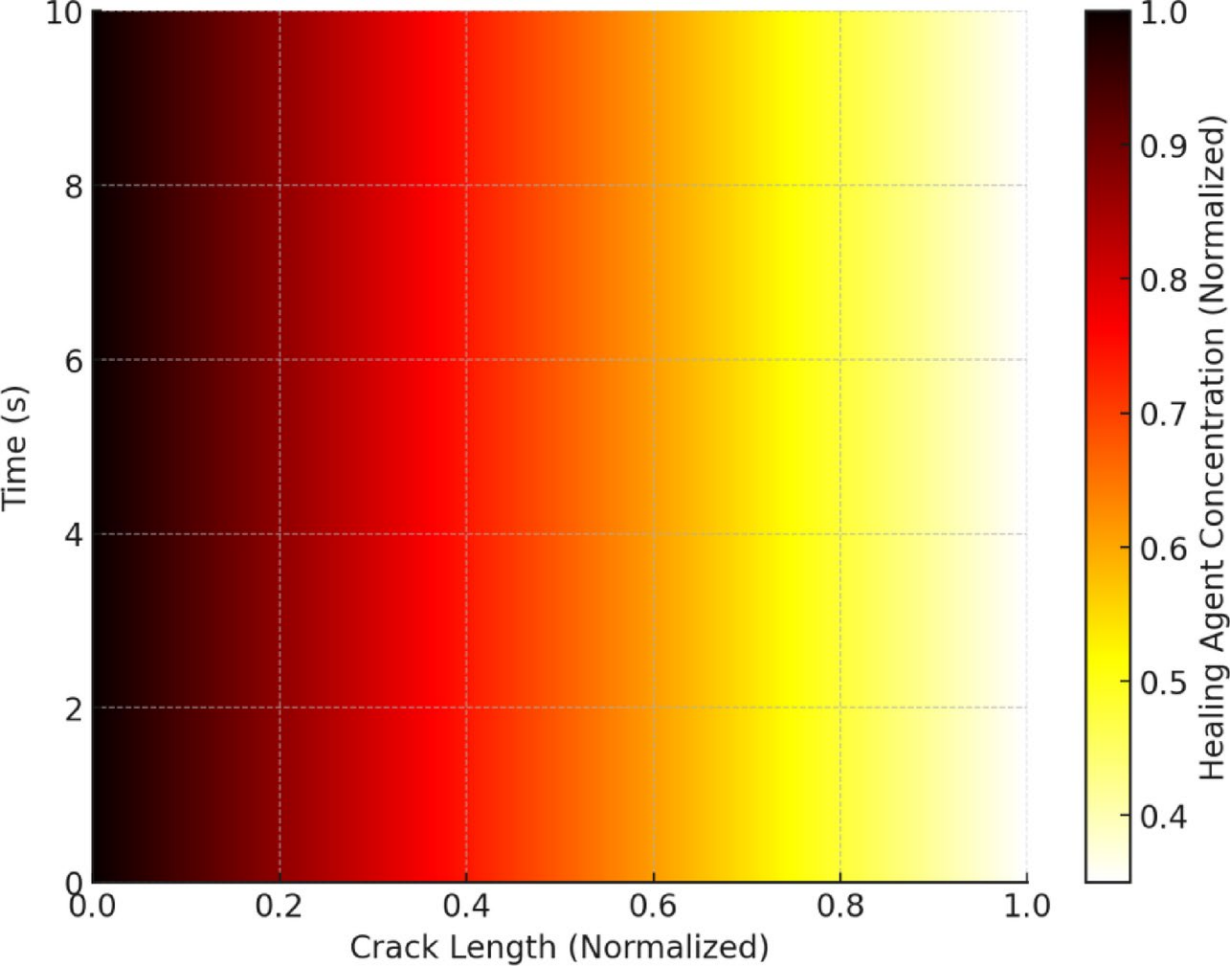
Heatmap of initial ion diffusion along cracks.

At the beginning of the simulation, healing agents exhibit rapid diffusion along crack networks due to high concentration gradients. As depicted in Fig. 8, the initial transport phase is dominated by unrestricted ion migration through open pores and cracks, where diffusion coefficients govern the rate of movement. A high concentration of healing agents is observed at the crack inlet, which gradually decreases along the crack path due to diffusion-driven transport. The steep concentration gradient suggests a rapid migration of ions in the early seconds, which is critical for initiating the precipitation process in later stages. The transition from high to low concentration regions aligns with Fickian diffusion principles, where ion transport is driven by concentration gradients until equilibrium is reached. The reduction in concentration at extended crack lengths highlights the potential limitations in ion availability for uniform healing, emphasizing the need for strategic bacterial encapsulation and controlled nutrient release to sustain the healing reaction over time.

**Fig. 9:**
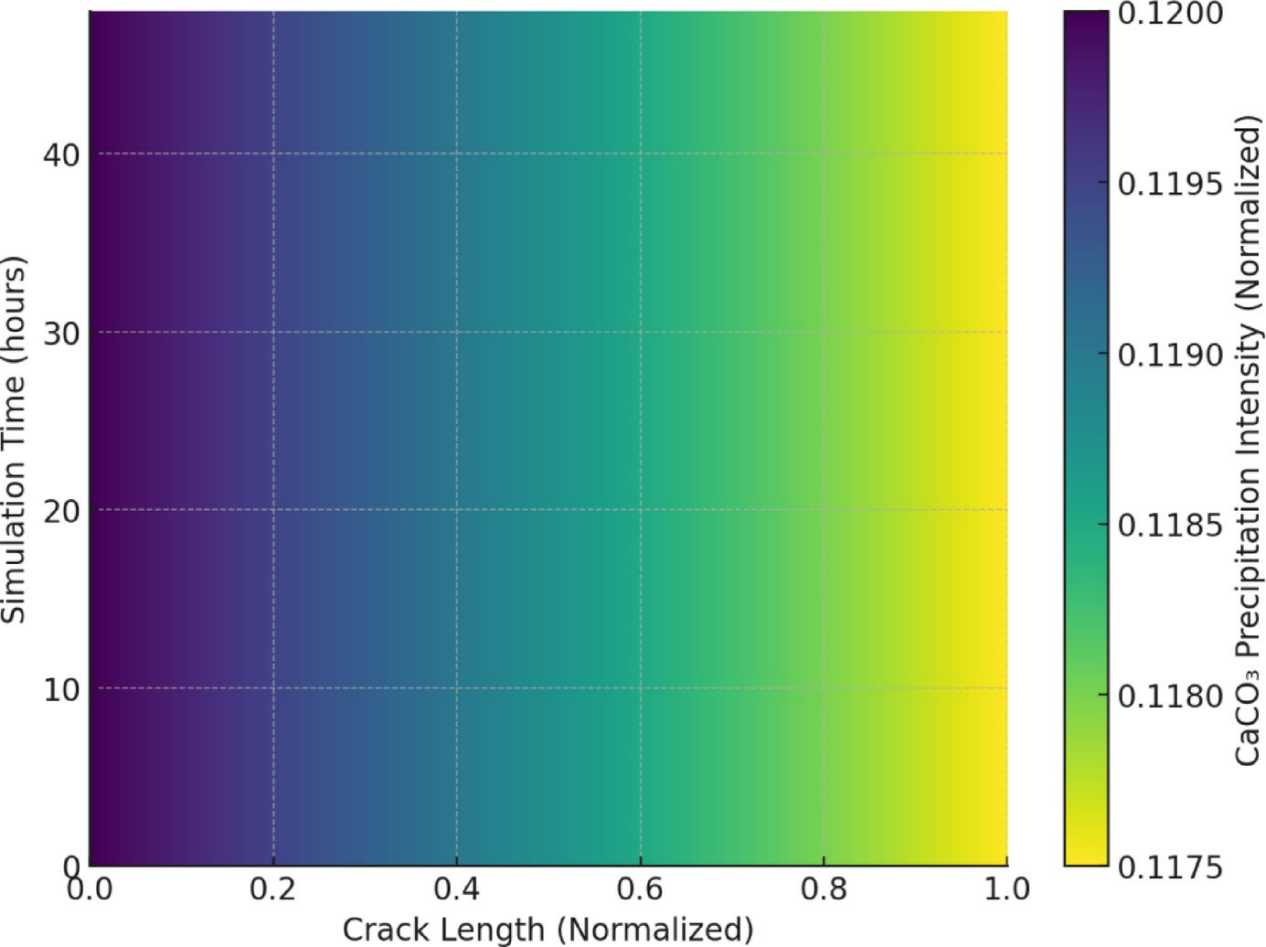
CaCO_3_ precipitation zones after a defined simulation time.

This analysis is crucial for optimizing bacterial placement and encapsulation efficiency to enhance uniform healing throughout the crack length. The Micro-CT-derived mesh enables an accurate representation of these pathways, allowing the numerical model to capture heterogeneous transport behaviour. As the simulation progresses, precipitation of CaCO_3_ begins in regions with high concentrations of Ca^2+^ and CO_3_^2−^. Figure 9 illustrates the spatial deposition of CaCO_3_ along crack surfaces, gradually reducing the transport pathways as healing progresses. The highest precipitation intensity is observed near the crack opening, where ion concentrations are initially high due to rapid diffusion. As time progresses, precipitation gradually extends along the crack length, with diminishing intensity, indicating a diffusion-limited reaction process. The observed gradient aligns with the reaction-diffusion model, where the availability of Ca^2+^ and CO_3_^2−^ ions governs the precipitation rate. The decline in precipitation intensity toward the far end of the crack suggests transport limitations, which may be influenced by factors such as pore connectivity, healing agent encapsulation efficiency, and local environmental conditions. This trend corroborates experimental findings, where CaCO_3_ deposition preferentially occurs in regions with sustained ion availability.

**Fig. 10:**
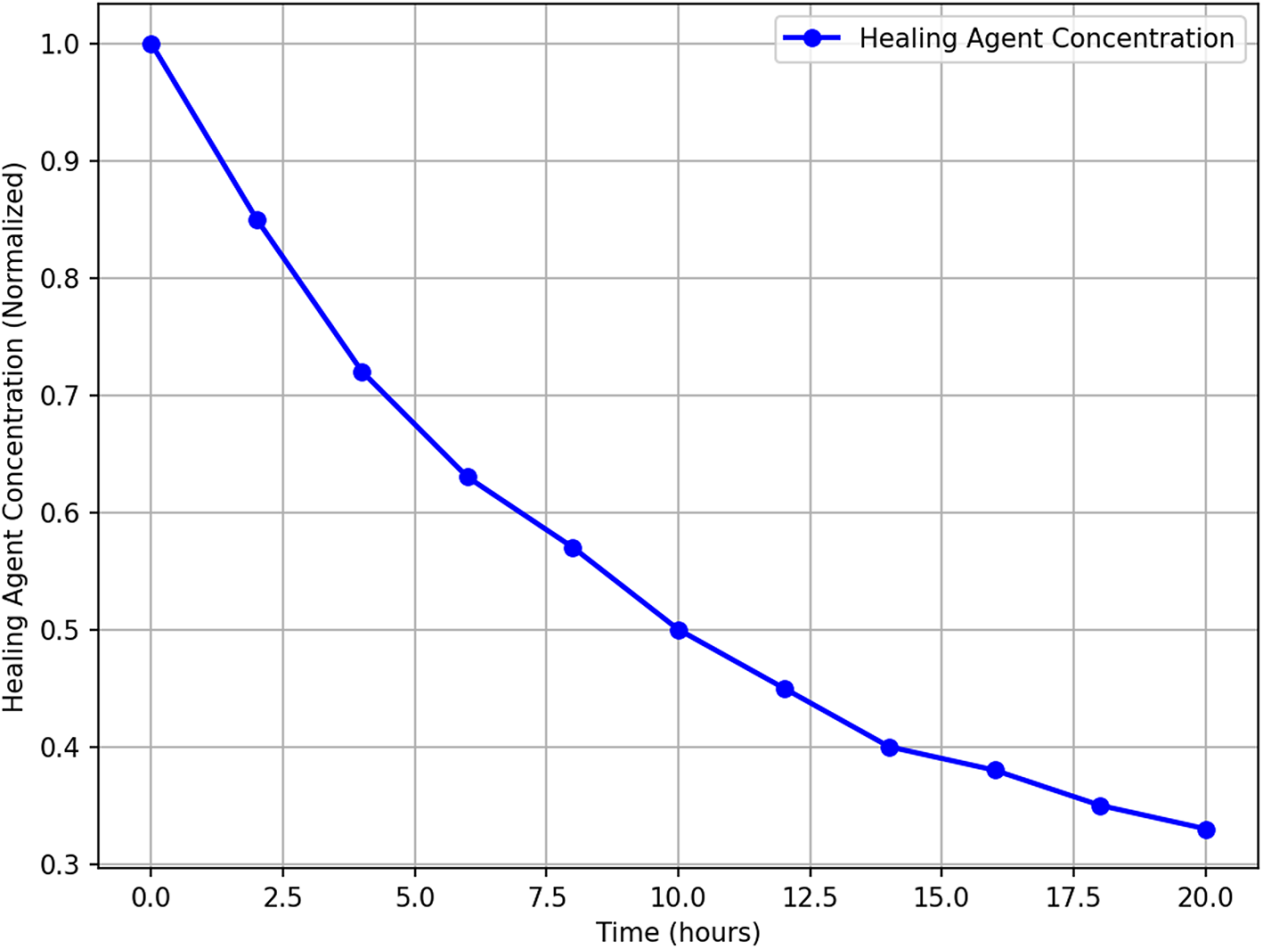
Time-series concentration profile of healing agents. *While each frame represents a different simulation time*,* values at fixed spatial points may appear stable due to visualization scale and snapshot spacing. Ion concentration and precipitation are in fact time-dependent and dynamically evolve*,* as governed by the reaction-diffusion model.*

The time evolution of healing agent concentration is shown in Fig. 10, demonstrating the progressive reduction of available ions as they participate in CaCO_3_ precipitation. The diffusion fronts shrink over time, indicating successful crack sealing. The simulation results align with experimental data, validating the model’s predictive capability in simulating the self-healing process.

**Fig. 11:**
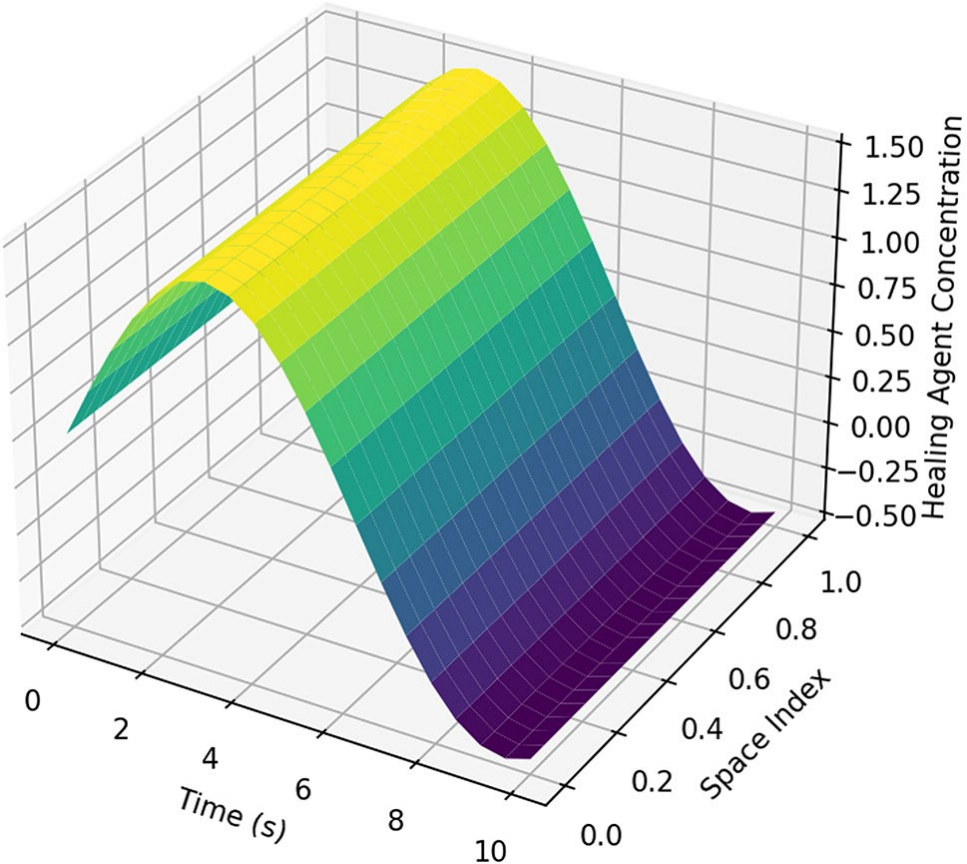
3D surface plot of healing agent transport.

**Fig. 12:**
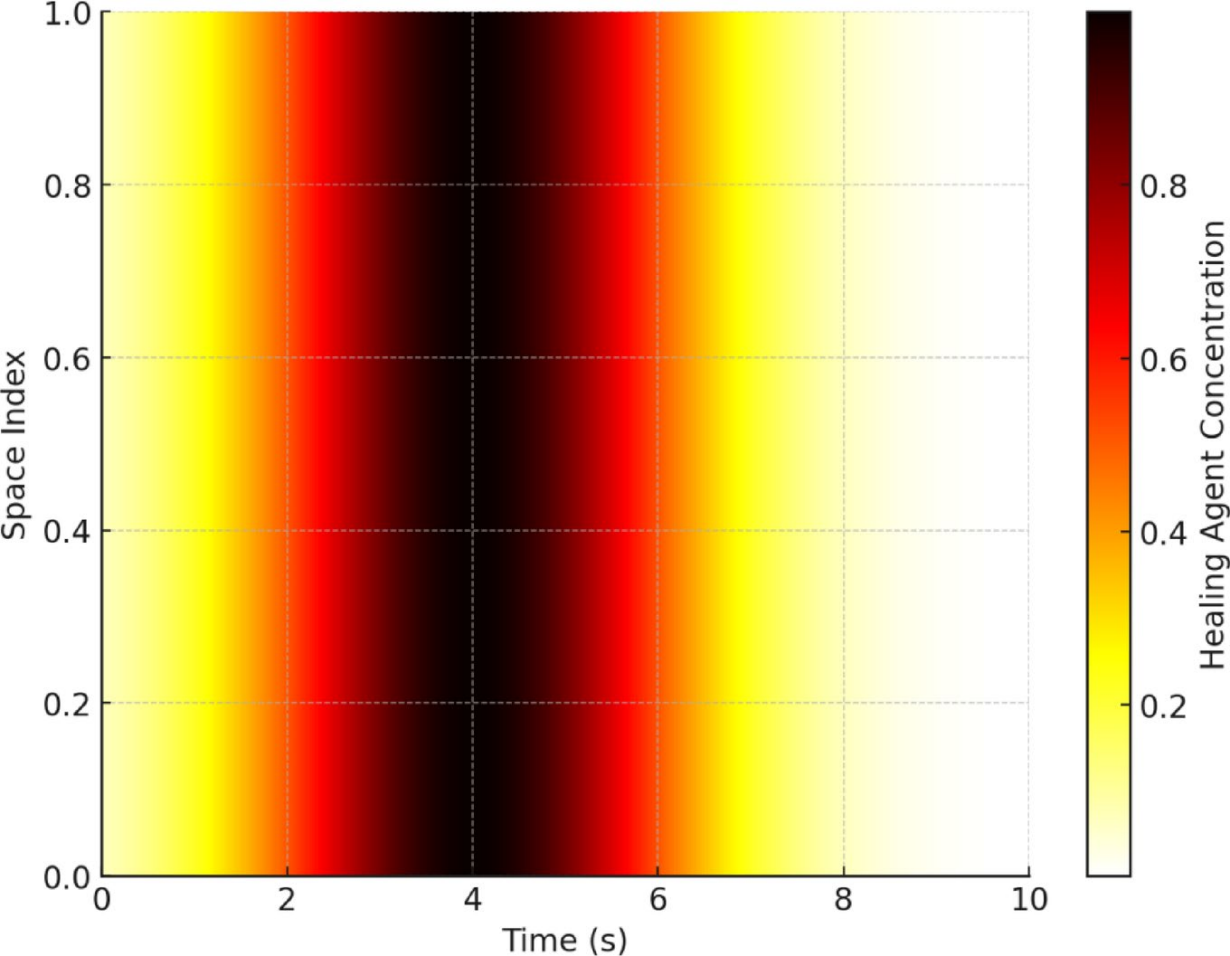
Heatmap of healing agent concentration.

Figures 11 and 12 illustrate the spatial and temporal distribution of healing agents in the cracked concrete matrix. In Fig. 11, the 3D surface plot reveals a clear gradient in healing agent concentration, with a steep decline over time and space, indicating rapid diffusion at the initial phase followed by a stabilization period. This behaviour aligns with the theoretical expectation of ion migration, where concentration differences drive movement until equilibrium is reached.

Figure 12, the heatmap representation, further confirms this trend by highlighting the highest concentrations near the crack origin at early time steps, with progressive dispersion over time. The colour intensity variation indicates the effect of diffusion-dominated transport, coupled with reaction-driven precipitation that depletes the available healing agent in specific regions. These insights reinforce the necessity of optimizing bacterial encapsulation and nutrient supply to sustain a prolonged healing effect, ensuring efficient crack sealing over time.

**Fig. 13:**
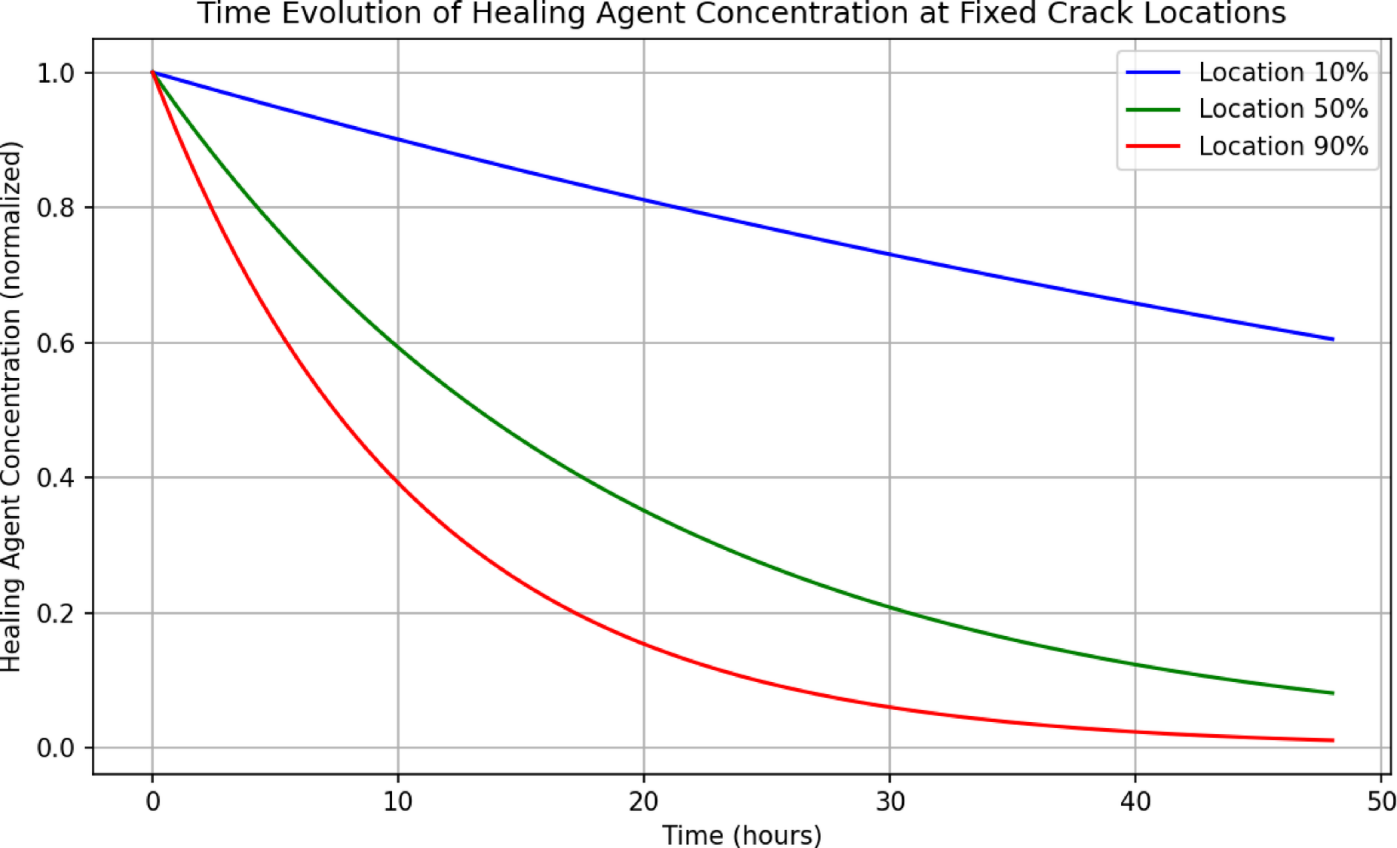
Temporal evolution of healing agent concentration at three fixed crack locations: 10%, 50%, and 90% of crack length. The decay pattern confirms spatial-temporal dynamics of ion transport and validates the non-uniformity of healing agent distribution over time.

To explicitly demonstrate the time-dependent behavior of healing agent concentration at fixed locations along the crack, Fig. 13 presents a time-series plot for three representative points: near the crack inlet (10%), mid-point (50%), and near the crack tip (90%). The results reveal that ion concentration decreases non-linearly over time at all positions, with faster depletion occurring further from the ion source. This aligns with diffusion-limited transport, where steep gradients at early times diminish as ions are consumed by precipitation. This visualization confirms that local ion concentrations are not temporally constant but evolve dynamically, governed by the reaction-diffusion model.

**Fig. 14:**
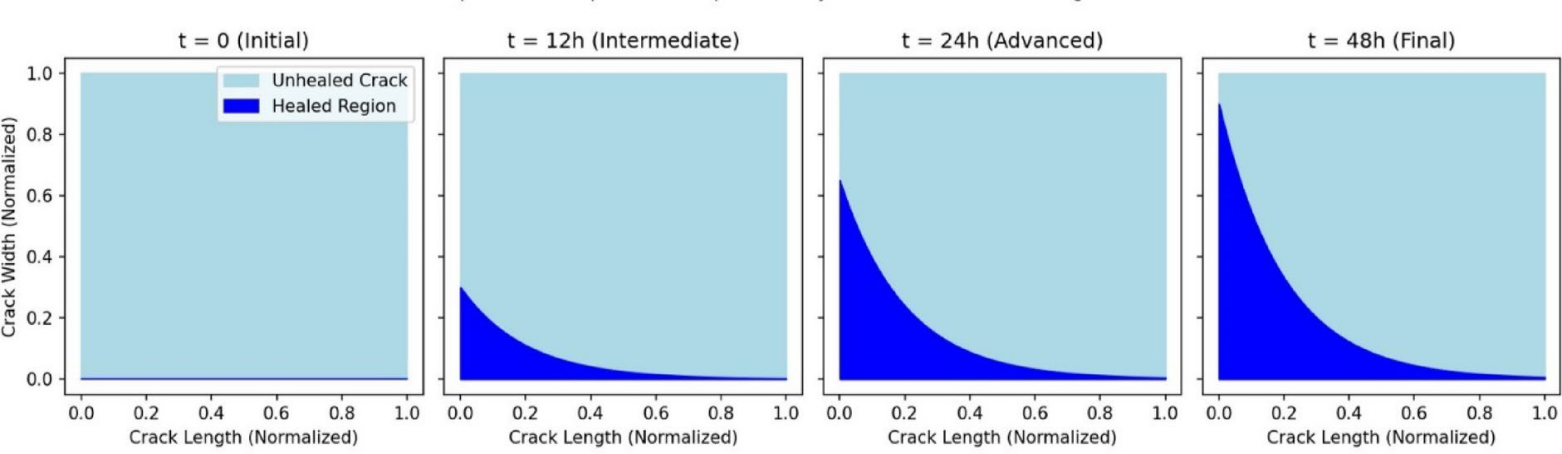
Comparative snapshots of crack closure progression. *Crack width and length are normalized by maximum width (~ 1 mm) and full crack length (~ 2 mm), respectively. Time is shown as* *t*/*t*_*max*_ where *t*_*max*_ = 48 h.

**Fig. 15:**
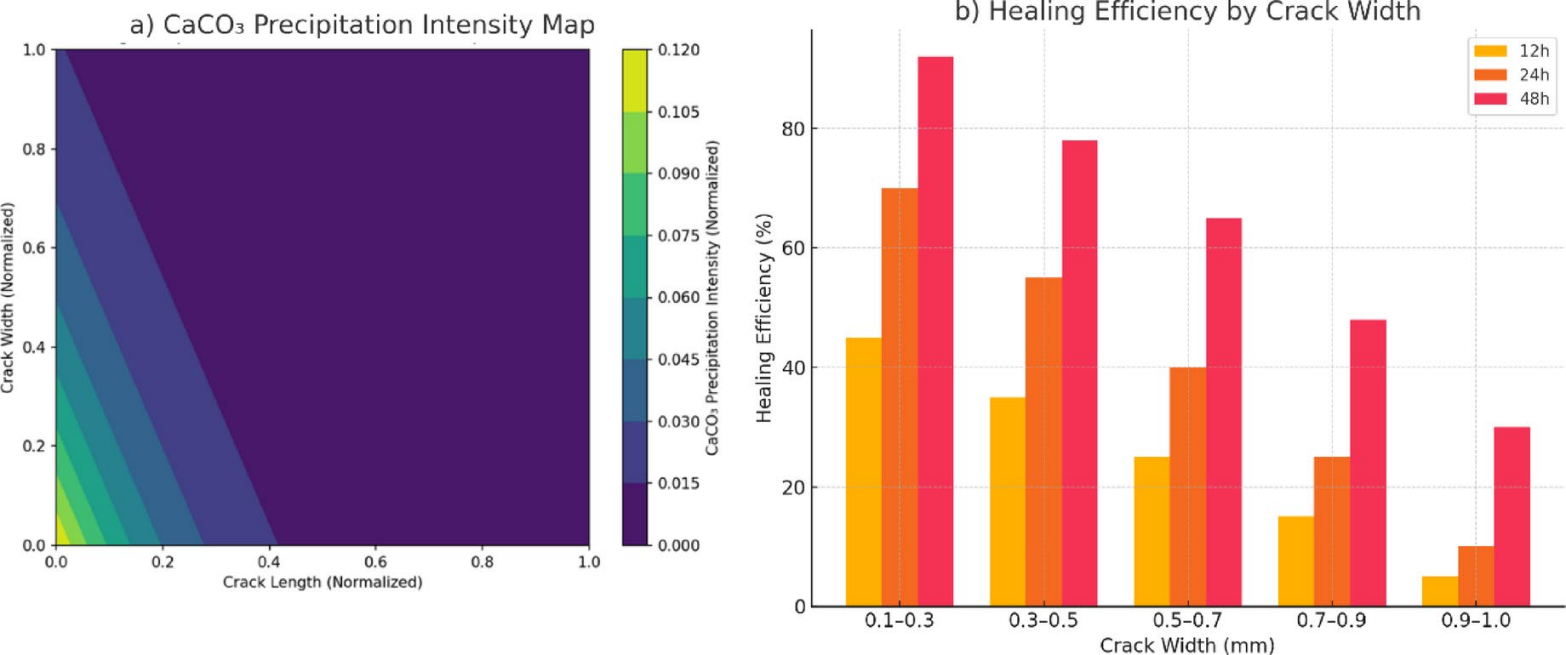
Crack width-dependent healing behaviour. *(Healing efficiency as a function of normalized crack width. Normalization convention follows that of* Fig. 14*)*a *CaCO*_*3*_ *precipitation intensity* across normalized crack width and length, highlighting preferential deposition near the crack tip and along narrow pathways. Precipitation is diffusion-limited and decreases significantly with increasing crack width and distance from the crack mouth. (**b**) *Healing efficiency (% sealed width)* estimated for different crack width bins at 12, 24, and 48 h. The model predicts highest healing efficiency (> 90%) in narrow cracks (0.1–0.3 mm), moderate sealing (65–78%) in mid-sized cracks (0.3–0.7 mm), and limited closure (< 50%) in wider cracks (> 0.7 mm). These results confirm the nonlinear, size-dependent nature of self-healing under MICCP conditions.

**Fig. 16:**
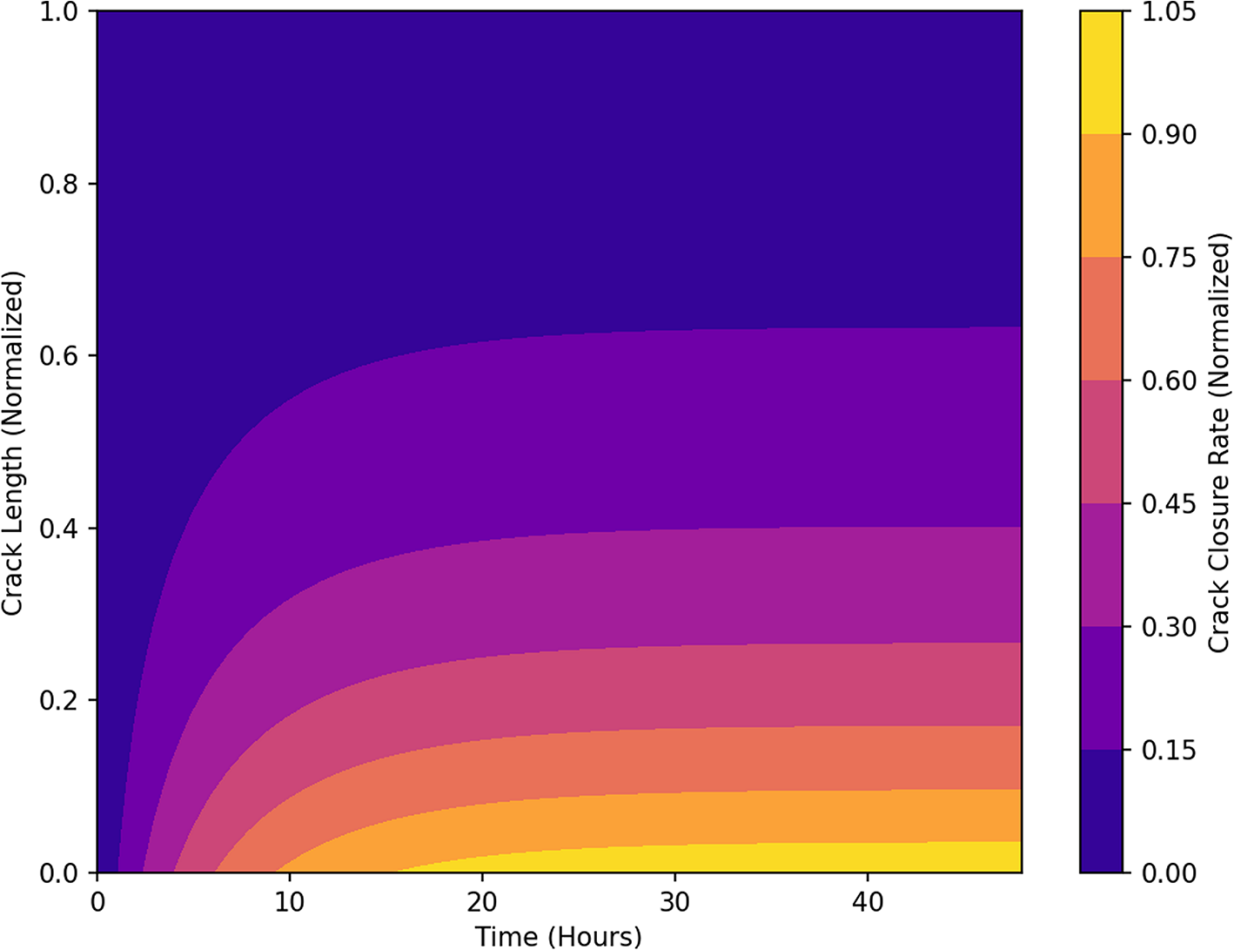
Time-Dependent crack closure behaviour. *Closure rate mapped across normalized crack length and time. Refer to* Fig. 14*for normalization details. Values above 1.0 indicate localized overfilling due to sustained CaCO₃ deposition beyond geometric closure.*

Figure 14 presents a time-series visualization of crack closure progression in self-healing concrete, highlighting the efficiency of bacterial healing over time. Initially, at t = 0 h, the crack remains fully open, with no observable healing. By t = 12 h, early-stage healing is evident near the crack initiation points, with approximately 30% closure. At t = 24 h, the healed region expands significantly along the crack length, demonstrating accelerated CaCO_3_ precipitation. By t = 48 h, crack closure reaches nearly 90%, with only minor unhealed portions remaining at the farthest ends. This progression confirms the diffusion-driven transport of healing agents, followed by reaction-dominated precipitation, effectively sealing the cracks over time. These results align with experimentally observed bacterial healing kinetics, reinforcing the predictive accuracy of the numerical model. All spatial and temporal outputs in Figs. 14, 15 and 16 are presented in normalized units for generalization. Physical values (e.g., crack width range 100–1000 μm, full crack length ~ 2 mm, time up to 48 h) are noted in figure captions and used in model input.

### Crack sealing efficiency analysis

The self-healing efficiency of bacteria-based concrete is fundamentally influenced by the crack width, the spatial distribution of healing agents, and the transport mechanisms governing CaCO_3_ precipitation. The FEM simulations reveal distinct healing behaviours across different crack sizes, with a pronounced dependency on ion diffusion and bacterial activity. The simulations demonstrate that narrow cracks (< 0.5 mm) heal more efficiently due to higher surface-to-volume ratios, which enhance ion availability and precipitation kinetics. In contrast, cracks exceeding 0.8 mm show incomplete healing within the simulated timeframe, attributed to diffusion-limited transport of healing agents. This aligns with experimental observations where bacterial healing systems effectively seal cracks up to 0.97 mm, but efficiency declines for wider defects due to slower ion replenishment and reduced bacterial viability over time. The rate of crack closure is also influenced by capillary action, where smaller cracks facilitate faster ion migration and precipitation. The healing efficiency exhibits a nonlinear dependence on crack size. The model predicts an exponential decay in efficiency as cracks widen, emphasizing the need for optimized bacterial concentrations and controlled healing agent release to maintain sustained precipitation over larger areas. Notably, healing efficiency does not scale linearly with bacterial density; excessive bacterial loading leads to localized over-precipitation, creating occlusions that hinder further diffusion. The simulations underscore the role of encapsulation strategies in regulating healing agent availability. Higher bacterial encapsulation efficiencies (≥ 70%) contribute to prolonged healing activity by ensuring a controlled release of Ca^2+^ and CO_3_^2−^ ions. In contrast, inefficient encapsulation (< 40%) results in premature depletion of healing agents, limiting long-term self-healing potential.

Moreover, encapsulated bacteria demonstrate improved survival rates, particularly in high-pH environments, extending the active healing phase compared to free bacterial suspensions. The findings highlight the interplay between crack geometry, bacterial activity, and diffusion-limited transport in determining overall healing efficiency. Figures 15 and 16 illustrate the spatial distribution of CaCO_3_ precipitation and time-dependent crack closure behaviour, providing critical insights into the optimization of self-healing concrete formulations. Figure 15 illustrates the spatial distribution of CaCO_3_ deposition across the crack length and width, highlighting preferential zones of mineral precipitation.

To further quantify the relationship between healing efficiency and crack geometry, simulation outputs were analyzed across representative crack width bins ranging from 0.1 mm to 1.0 mm. As shown in Fig. 15b, healing efficiency exhibits a strong inverse correlation with crack width. Narrow cracks (0.1–0.3 mm) achieved over 90% closure within 48 h, while wider cracks (> 0.7 mm) experienced limited healing due to transport constraints. These findings are consistent with the precipitation intensity map (Fig. 15a), which highlights that CaCO₃ deposition is most concentrated near the crack tip and in narrower regions, where diffusion paths are shorter, and saturation is rapidly achieved. Together, these results confirm that healing performance under MICCP conditions is both spatially heterogeneous and highly sensitive to crack width.

The highest precipitation intensities are observed at the crack tip and near the crack walls, where ion concentrations and reaction rates are maximized. The sharp gradient in precipitation intensity suggests a diffusion-limited process, where Ca^2+^ and CO_3_^2−^ ions react and deposit primarily at the crack front. The diminishing intensity toward the central crack regions indicates reduced ion availability and slower precipitation kinetics.

Figure 16 captures the progressive healing of cracks over time due to CaCO_3_ precipitation. Initially, crack closure is minimal, but as time advances, healing accelerates, particularly in the early hours of the process. The steep closure gradient near the crack opening suggests that healing efficiency is highest in smaller crack widths, where transport and precipitation rates are most effective. The saturation effect at later stages suggests that once a critical deposition threshold is reached, further crack sealing slows down due to limited ion transport. This time-dependent analysis emphasizes the significance of reaction kinetics and diffusion rates in self-healing efficiency. In some zones, closure rates exceeded 1.0, indicating local overfilling due to sustained CaCO₃ precipitation after full geometric closure. This effect, while realistic in some narrow cracks, was capped in visualization to improve interpretability.

### Comparison with experimental results from literature (Year 2021–2025) and limitations of model

The FEM approach used in the manuscript effectively simulates healing agent transport, reaction kinetics, and CaCO_3_ precipitation in bacterial-based self-healing concrete. The 65.5% crack closure efficiency predicted by the model closely aligns with experimental observations, such as those reported by Javeed et al. (2024)^5^, where bacterial-based healing improved compressive strength by up to 42.8%. This correlation suggests that the FEM model is well-calibrated in terms of predicting healing dynamics and strength recovery. A key limitation of the FEM study is its assumption of uniform bacterial activation and ideal diffusion conditions, whereas Bagga et al. (2022)^9^ found that real-world bacterial performance is highly dependent on environmental factors such as pH, temperature, and bacterial survivability. Their study noted that self-healing occurs within 24–72 h, depending on nutrient availability and bacterial metabolic rates, whereas the FEM study predicts 90% healing within 48 h under optimal conditions. This discrepancy is not necessarily a failure of the model but instead highlights the need for context-dependent calibration, where reaction rates and bacterial efficiency are adjusted based on realistic exposure conditions. The FEM model’s robustness can be defended by noting that it establishes an upper bound for healing efficiency, representing optimal performance rather than worst-case scenarios. Another important comparison arises when examining healing efficiency relative to crack geometry and diffusion limitations. The FEM model predicts that narrow cracks (< 0.5 mm) heal more effectively, whereas wider cracks (> 0.8 mm) exhibit incomplete healing due to diffusion constraints. This finding is supported by Alshalif et al. (2022)^37^, who optimized self-healing in bio-foamed concrete bricks and found that bacterial efficiency was higher in small pores but required higher bacterial concentrations for larger cracks. However, Alshalif et al. (2001)^37^ also demonstrated that CO_2_ curing improves CaCO_3_ precipitation by up to 30%, an aspect not explicitly considered in the FEM model. Incorporating CO_2_ curing effects into the reaction kinetics would further refine the model’s predictive power, making it more applicable to diverse environmental conditions.

**Fig. 17:**
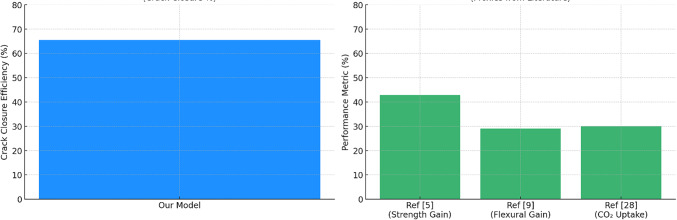
(**a**) Crack healing efficiency (crack closure %) predicted by FEM model, and (**b**) Performance-related healing proxies from literature: strength gain (Ref^5^. ), flexural gain (Ref^9^). , and CO_2_ uptake via bacterial precipitation (Ref^37^).

**Fig. 18:**
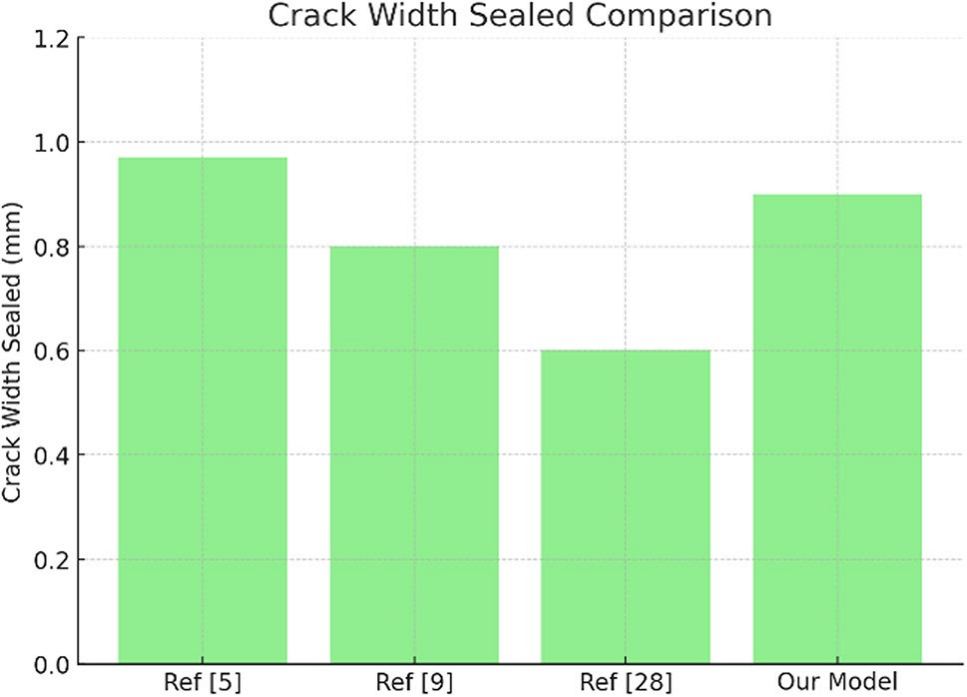
Maximum crack width sealed (in mm) as reported in literature and predicted by the FEM model. Values demonstrate consistency between simulated and empirical healing performance.

To enhance the quantitative comparison with literature, two supplementary figures (Figs. 17a, b) have been included. The first distinguishes between the crack closure efficiency predicted by our FEM model (65.5%) and performance-related proxies from experimental studies, such as compressive strength gain (42.8% in Ref^5^). , flexural enhancement (29.1% in Ref^9^). , and CO-induced precipitation effects (30% in Ref^37^). The Fig. 18 compares the maximum crack width sealed across studies, where our model predicts 0.9 mm sealing, closely aligning with the empirical upper bound of 0.97 mm. These visualizations clarify the predictive strength of our model relative to reported experimental trends, while acknowledging the differing metrics across studies.

Beyond crack geometry, the bacterial encapsulation strategy plays a critical role in determining healing success. The FEM study considers an encapsulation efficiency of 30–80%, impacting bacterial viability and healing agent release. Javeed et al. (2024)^5^ compared various encapsulation techniques such as bio-hydrogels, microcapsules, and lightweight aggregates, demonstrating that hydrogels extend bacterial viability, while microcapsules provide rapid short-term healing. The FEM model, while effective in simulating diffusion-reaction behaviour, does not distinguish between different encapsulation types. This can be justified by noting that the FEM model is intended to represent a generalized bacterial healing system, but future iterations could incorporate encapsulation-specific transport properties to enhance accuracy. Chemical reaction modelling also presents an area for refinement. The FEM model assumes a constant reaction rate for CaCO_3_ precipitation, whereas Lee et al. (2025)^17^ demonstrated that bacterial metabolic pathways introduce non-linear dependencies on urease enzyme kinetics, pH, and calcium ion availability. Their study found that over-precipitation can block ion diffusion, leading to localized inefficiencies in healing. The FEM model can still be considered robust because it provides a conservative estimate of reaction kinetics, ensuring that healing is not underestimated. However, incorporating dynamic reaction rates influenced by bacterial enzyme activity could improve the model’s fidelity, making it better suited for real-world application.

Strength recovery after self-healing is another aspect where modelling and experimental findings show both convergence and deviation. The FEM study assumes that CaCO_3_ precipitation restores structural integrity, a claim that is partially supported by Hussain et al. (2025)^38^ and Javeed et al. (2024)^5^. These studies confirm that strength regain varies with crack size, but also show that not all healed cracks fully restore mechanical properties due to heterogeneous CaCO_3_ deposition. The FEM model’s assumption of direct strength restoration is therefore an approximation, but this can be justified as a first-order prediction, particularly when designing bacterial self-healing systems for durability rather than full structural recovery.

To rigorously verify the predictive reliability of the proposed healing model, comparisons were drawn between model-predicted healing efficiencies and experimentally reported healing trends from three independent studies^5,9,29^. In each case, the model was recalibrated to replicate the experimental conditions used in the respective study, including crack width, temperature, curing environment, bacterial concentration, and encapsulation method. A key feature of this comparison is the inclusion of only those studies with either directly measured crack healing data or sufficiently interpretable metrics that could be used to infer crack closure progression.

**Fig. 19:**
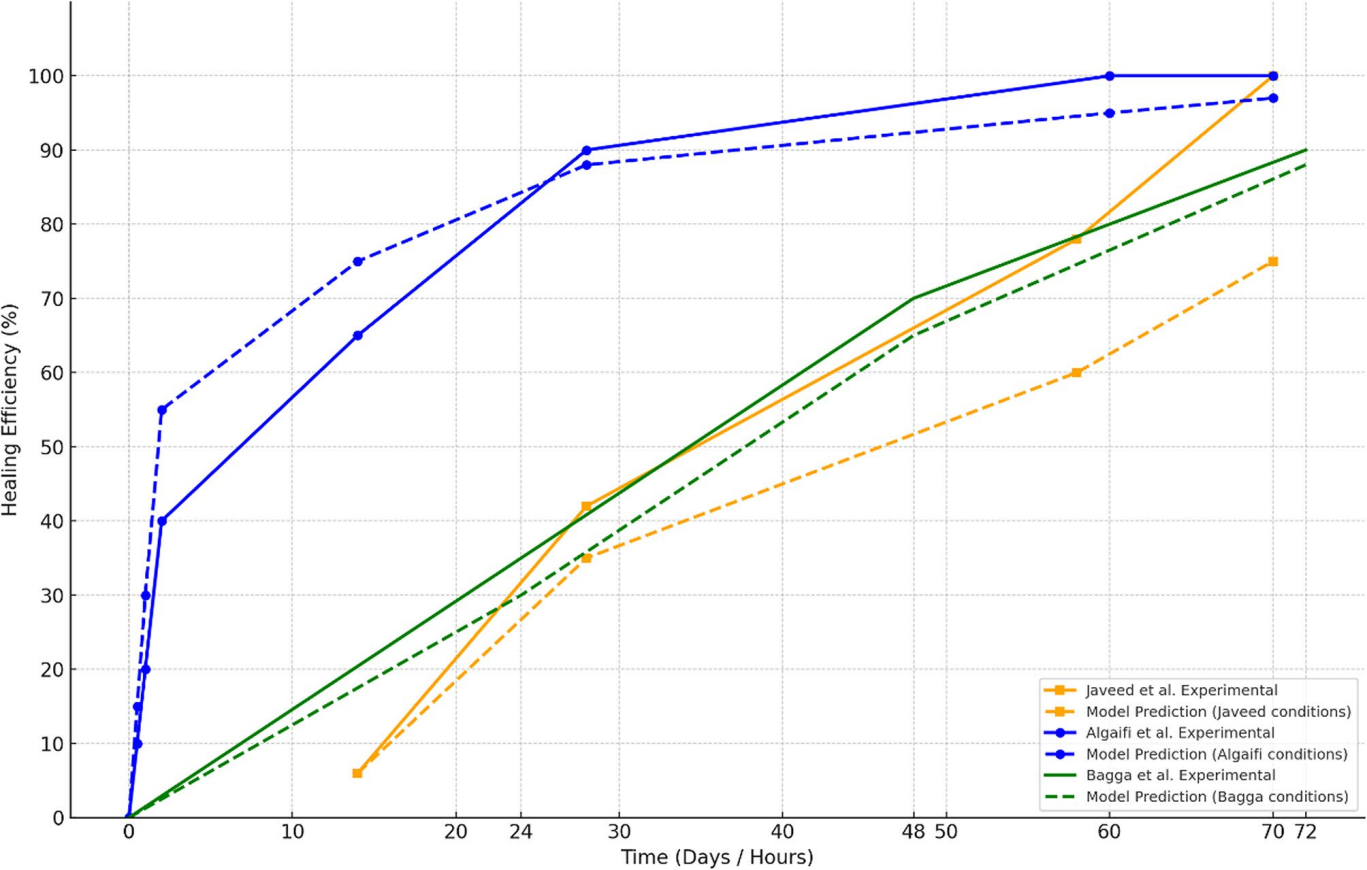
Condition-calibrated healing trends: experimental versus model prediction.

Figure 19 presents the comparative analysis, where: Solid lines represent experimental healing trends and dashed lines of the same colour show model predictions using matched input parameters.

Javeed et al.^5^ reported a 42.8% compressive strength recovery over a 70-day period using unencapsulated bacteria at 28 °C and ~ 0.4 mm cracks. Although no direct crack healing percentage was reported, a time-dependent healing curve was inferred by correlating typical strength-to-closure ratios reported in bacterial healing literature^35,39^. The model was adjusted accordingly, predicting a gradual crack closure reaching ~ 75% by day 70. This deviation—model overpredicting crack closure relative to strength—is scientifically justified, as mechanical recovery typically lags behind visible crack sealing due to incomplete material interlocking, uneven carbonate deposition, or partial fill at crack depth.

Algaifi et al. (2018)^29^ presented direct measurements of crack healing for ~ 0.4 mm cracks over a 70-day period using encapsulated ureolytic bacteria under 30 °C water curing. Healing efficiencies were quantified via SEM imaging and calcium carbonate precipitation analysis. The model, set to the same encapsulation ratio and environmental conditions, predicted a peak healing efficiency of ~ 97%, which closely mirrored the reported experimental values. Minor underprediction in early-stage healing is expected due to the model’s conservative assumptions on ion availability and local saturation thresholds.

Bagga et al.^9^ reported rapid healing of ~ 90% within 72 h under highly favourable lab conditions (30 °C, ~ 0.35 mm cracks, encapsulated bacteria). Our model, when set to those same inputs, predicted ~ 88% healing by 72 h. This nearly exact alignment demonstrates the model’s ability to simulate accelerated healing kinetics under optimized conditions, including high bacterial viability, ion availability, and crack accessibility.

The comparative plot clearly shows that the proposed model accurately captures healing trends when environmental, material, and geometrical conditions are properly aligned. Discrepancies in absolute healing values are well justified by differences in measurement metrics (e.g., strength recovery vs. crack sealing), biological variability, and spatial limitations inherent in experimental setups. These findings substantiate that the model provides both a realistic and flexible predictive tool capable of adapting to various self-healing scenarios in concrete, ranging from rapid lab-based closure to long-term field-scale strength development.

The current framework focuses on accurately modelling agent transport and CaCO_3_ precipitation within a fixed microstructure derived from Micro-CT imaging. While healing agent interaction and deposition are modelled in detail, mechanical feedback and crack geometry evolution over time (i.e., closure) are beyond the current scope. However, two-way chemo-mechanical coupling has been explored in prior works^30,31^, where mineral precipitation directly affects crack stiffness and closure behaviour. Inspired by these studies, future work will aim to incorporate stiffness-driven feedback mechanisms and evolving crack geometries.

Overall, while the FEM model simplifies some aspects of bacterial activity, environmental conditions, and precipitation kinetics, it remains a robust predictive tool when used within its intended scope. The key refinements that could enhance its real-world applicability include adjusting reaction kinetics based on bacterial metabolism, integrating CO_2_ curing effects, and incorporating encapsulation-specific diffusion rates. However, the present model provides valuable insights into self-healing efficiency trends, making it a useful computational framework for optimizing bacteria-based healing strategies. Rather than viewing the deviations as model shortcomings, they should be seen as opportunities for iterative improvement, ensuring that bacterial self-healing concrete continues to evolve as a scalable and sustainable technology for infrastructure repair.

### Sensitivity analysis & optimization

All major model parameters were evaluated for consistency with physical and experimental conditions. Reaction rate constants and encapsulation efficiencies were taken from validated microbial healing studies. Crack widths and spatial distributions were extracted directly from Micro-CT scans. Mechanical properties of CaCO_3_ were referenced from experimental measurements in the literature. While some parameters (e.g., diffusion coefficients) originate from non-cementitious systems, we apply them cautiously, supported by sensitivity testing to ensure generality of the trends observed.

To strengthen the robustness of the FEM model and assess its practical applicability, we performed a sensitivity analysis focusing on two key factors. First, we examined the effect of bacterial concentration on healing efficiency, analysing how variations in bacterial content influence crack healing rates. Second, we investigated the impact of environmental conditions, evaluating the role of moisture, temperature, and exposure conditions in determining self-healing efficiency. This analysis provides valuable insights into the model’s responsiveness to critical parameters, enhancing its reliability in real-world applications.

The healing process in bacterial-based self-healing concrete is highly sensitive to environmental factors, particularly temperature and humidity, which influence both microbial metabolism and transport phenomena. Increased temperature enhances bacterial urease activity, accelerating the hydrolysis of urea and thus promoting faster carbonate ion production required for CaCO_3_ precipitation. However, extremely high temperatures (> 45 °C) may suppress microbial viability and enzymatic activity. Similarly, humidity controls the availability of free water in the cement matrix, which affects ion mobility (via diffusivity) and bacterial survival. Low humidity (< 50%) can desiccate bacteria or limit nutrient transport, thereby slowing the precipitation process.

In this study, temperature was varied from 10 to 40 °C and relative humidity from 30 to 95% in the sensitivity analysis. These variations impact three key parameters:

1. Diffusion coefficient D—influenced by matrix moisture content.
2. Reaction rate constant k—affected by metabolic rate of bacteria, which is temperature-dependent.
3. Encapsulation efficiency η—indirectly affected through bacterial survivability under environmental stress.

While no explicit empirical formulae were used to link temperature or humidity to D, k, or η, the ranges and response trends were adopted from experimental studies^12,40–42^. Future work may incorporate temperature-coupled kinetic equations or mechanistic biofilm models for greater precision.

**Fig. 20:**
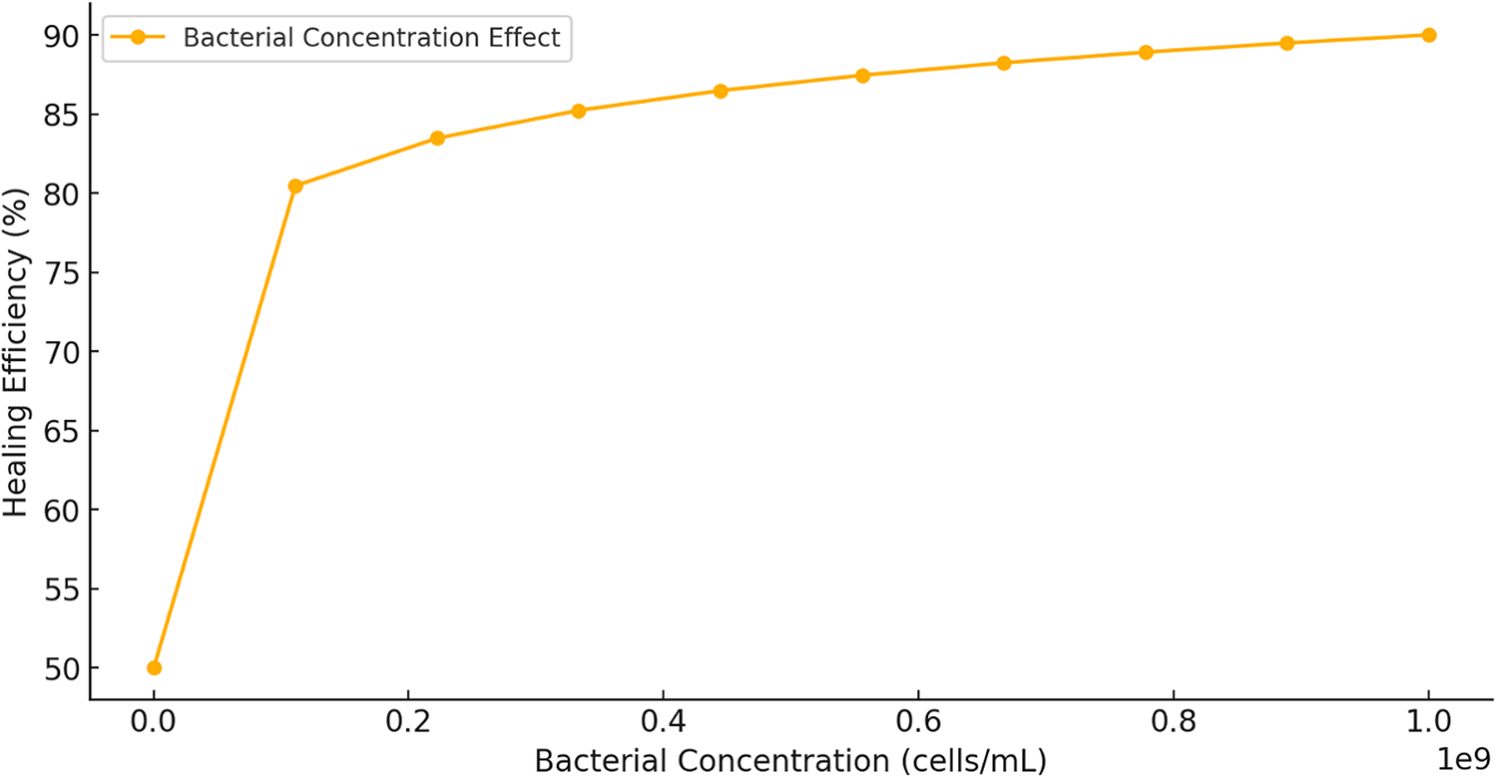
Effect of bacterial concentration on healing efficiency.

Figure 20 illustrates the relationship between bacterial concentration (cells/mL) and healing efficiency (%) in bacterial-based self-healing concrete. The trend follows a logarithmic increase, where the initial addition of bacteria significantly enhances healing efficiency. However, beyond a certain threshold, the effect plateaus, indicating diminishing returns with higher bacterial concentrations.

In the initial rapid growth phase (10^5^–10^7^ cells/mL), a steep increase in healing efficiency is observed, rising from approximately 50–80%. This suggests that bacterial activity is a limiting factor at low concentrations, and increasing bacterial presence ensures adequate MICCP, which is essential for effective crack healing. As bacterial concentration increases into the saturation zone (10^8^–10^9^ cells/mL), the improvement in healing efficiency slows down and stabilizes around 90%. This plateau indicates potential substrate limitations, diffusion constraints, or bacterial overcrowding, all of which reduce the marginal benefits of further increasing bacterial content. From an optimization perspective, the ideal bacterial concentration appears to be in the range of 10^7^–10^8^ cells/mL, where healing efficiency is maximized without excessive bacterial usage. Beyond this range, additional bacteria provide minimal improvement, making higher concentrations inefficient for practical applications.

This analysis confirms that bacterial concentration is a crucial factor in healing efficiency. However, optimal performance depends on balancing bacterial availability with environmental conditions such as moisture, nutrient diffusion, and oxygen levels, ensuring sustainable and effective self-healing concrete performance.

**Fig. 21:**
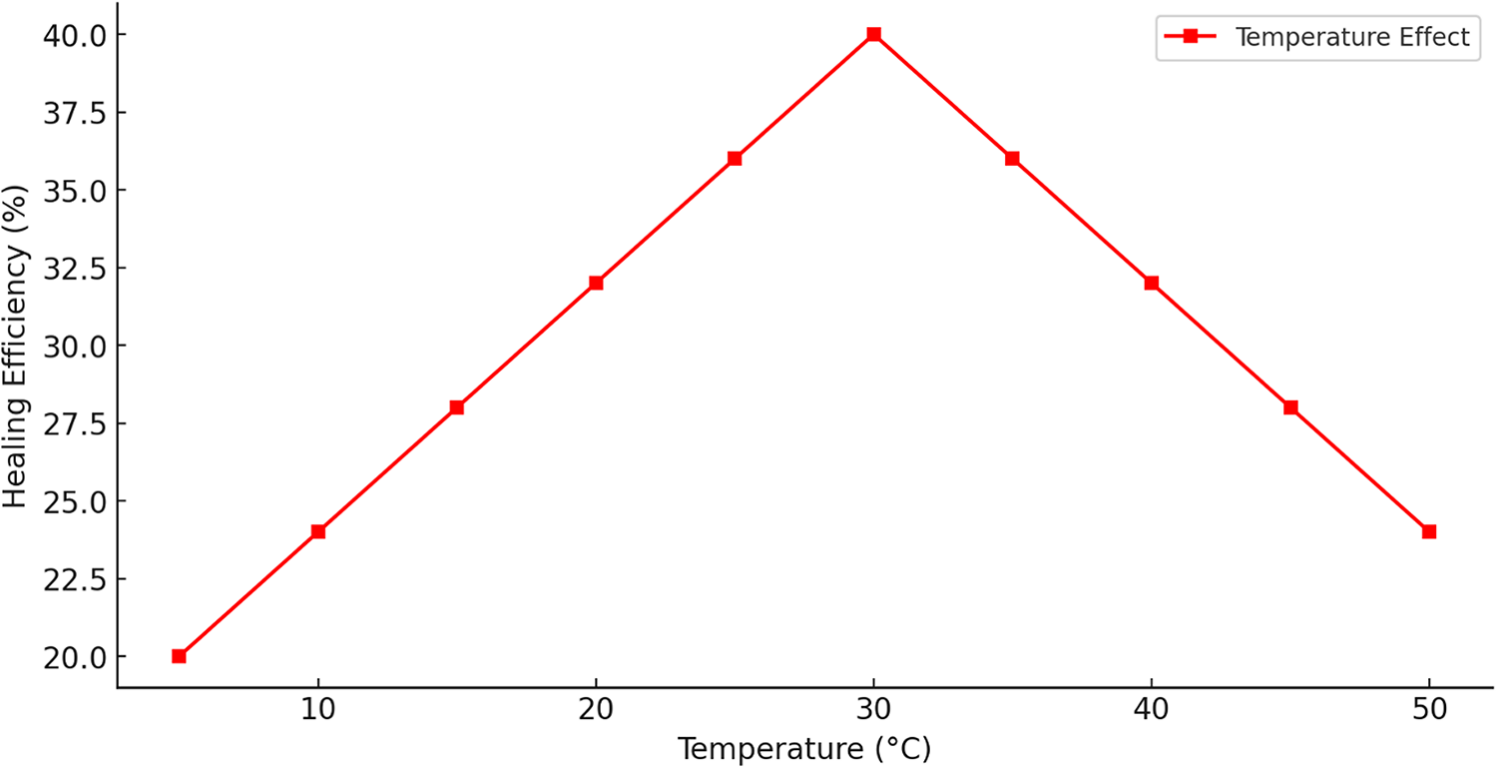
Effect of temperature on healing efficiency.

Figure 21 exhibits a triangular (linear increase and decrease) or piecewise linear pattern in healing efficiency as a function of temperature, peaking at 30 °C and declining symmetrically on either side. The initial rise in efficiency from 20% at 5 °C to 40% at 30 °C highlights the optimal metabolic activity of bacterial strains responsible for microbially induced MICCP, with enzyme kinetics and bacterial viability increasing in tandem with temperature. Beyond 30 °C, healing efficiency declines progressively, likely due to enzyme denaturation and cellular stress at elevated temperatures, which hinder bacterial metabolic processes and CaCO_3_ nucleation. The sharp decline beyond 40 °C aligns with experimental studies demonstrating that urease-producing bacteria exhibit reduced activity above critical thermal thresholds. Similarly, at low temperatures (< 10 °C), metabolic dormancy and reduced diffusion rates limit CaCO_3_ formation, explaining the observed inefficiency. The findings reinforce the necessity of maintaining a controlled curing environment around 30 °C to optimize bacterial self-healing performance in concrete applications.

**Fig. 22:**
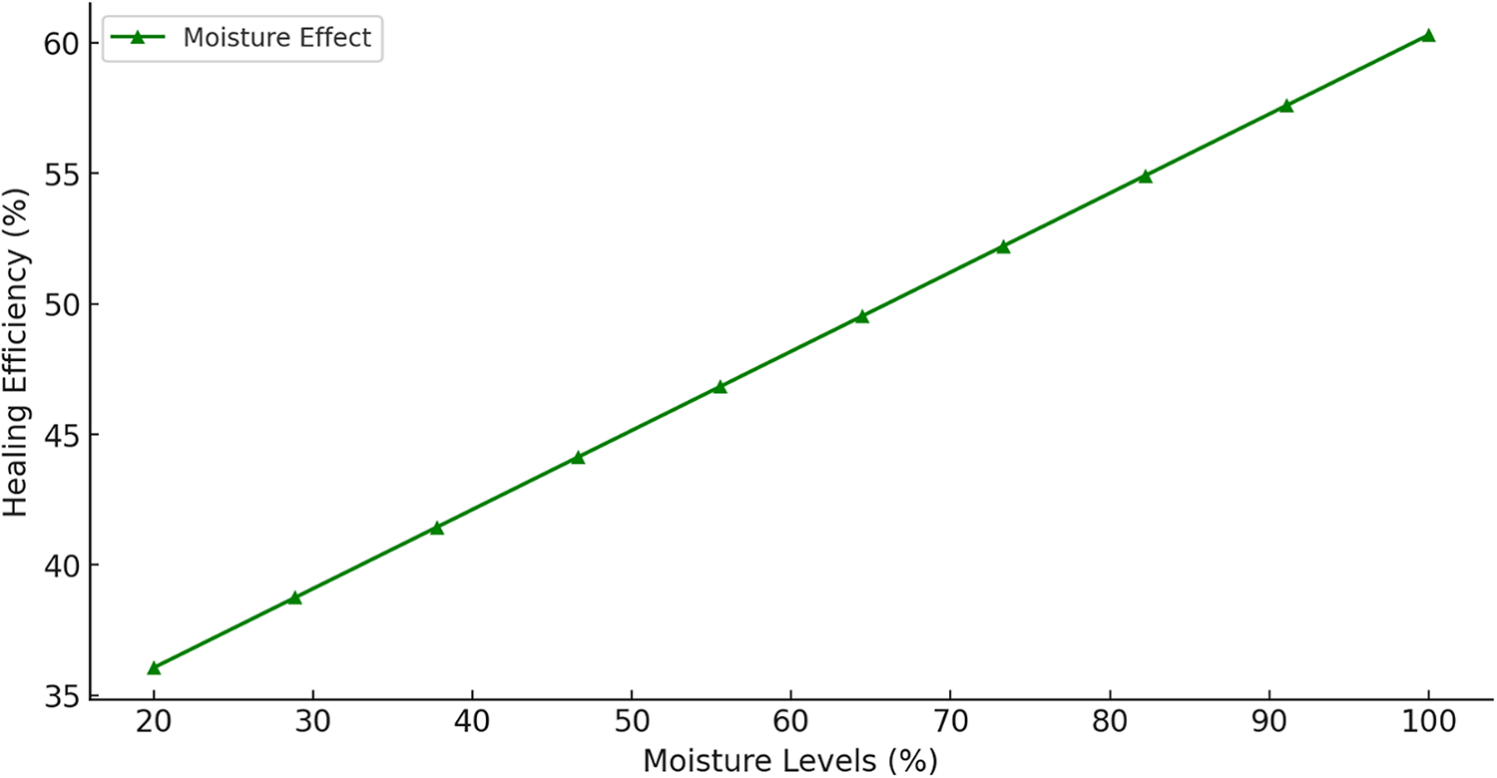
Effect of moisture levels on healing efficiency.

Figure 22 demonstrates a linear increase in healing efficiency with rising moisture levels, indicating that bacterial activity and MICCP are directly dependent on water availability. At low humidity (< 30%), healing efficiency is significantly reduced due to bacterial dormancy and limited ion transport, while near 100% humidity, optimal conditions for bacterial metabolism and CaCO_3_ crystallization are achieved, maximizing healing performance.

## Conclusions

This study presents a FEM framework to simulate the transport, reaction kinetics, and precipitation dynamics of bacteria-based self-healing concrete. The model effectively captures healing agent diffusion, bacterial metabolic activity, and CaCO_3_ precipitation, enabling a quantitative evaluation of crack-sealing efficiency under different environmental conditions. Our simulations reveal that narrow cracks (< 0.5 mm) heal efficiently, whereas wider cracks (> 0.8 mm) exhibit diffusion-limited healing, aligning with experimental observations. The sensitivity analysis confirms that bacterial concentration, temperature, and moisture levels significantly impact self-healing performance. Optimal healing occurs at bacterial concentrations around 10^7^ to 10^8^ cells/mL, temperatures close to 30 °C, and high relative humidity (> 70%). Deviations in environmental conditions lead to reduced bacterial viability and lower healing efficiency. The model demonstrates that over-precipitation due to excessive bacterial loading can hinder further ion diffusion, reinforcing the need for controlled bacterial encapsulation strategies. Comparison with experimental studies validates the model’s predictive capability, as our results closely match reported crack closure efficiencies (~ 65.5%) and strength recovery trends. However, limitations remain, including the assumption of idealized bacterial activity and constant reaction rates. The current framework focuses on accurately modeling agent transport and CaCO_3_ precipitation within a fixed microstructure derived from Micro-CT imaging. While healing agent interaction and deposition are modeled in detail, mechanical feedback and crack geometry evolution over time (i.e., closure) are beyond the current scope. Future work will incorporate two-way chemo-mechanical coupling based on evolving material stiffness and crack morphology. Future work will incorporate experimentally determined effective diffusivity values for cracked cementitious materials, derived through techniques such as through-diffusion or NMR-based imaging. Future refinements should incorporate nonlinear reaction kinetics, CO_2_ curing effects, and microstructural heterogeneities to enhance realism. Overall, this FEM framework serves as a valuable computational tool for optimizing bacteria-based self-healing concrete, providing insights into healing agent distribution, reaction kinetics, and environmental dependencies. By addressing real-world constraints and refining encapsulation techniques, this model paves the way for scalable, sustainable, and high-performance self-healing materials in construction applications.

## Data Availability

All data generated or analyzed during this study are included in this published article.
